# Metagenomes of the Picoalga *Bathycoccus* from the Chile Coastal Upwelling

**DOI:** 10.1371/journal.pone.0039648

**Published:** 2012-06-22

**Authors:** Daniel Vaulot, Cécile Lepère, Eve Toulza, Rodrigo De la Iglesia, Julie Poulain, Frédéric Gaboyer, Hervé Moreau, Klaas Vandepoele, Osvaldo Ulloa, Frederick Gavory, Gwenael Piganeau

**Affiliations:** 1 UPMC (Paris-06) and CNRS, UMR 7144, Station Biologique, Place G. Tessier, Roscoff, France; 2 CNRS and UPMC (Paris-06) UMR 7232, Oceanological Observatory of Banyuls (OOB), Banyuls sur mer, France; 3 Departamento de Oceanografía and Centro de Investigación Oceanográfica en el Pacífico Sur-Oriental (COPAS), Universidad de Concepción, Concepción, Chile; 4 Genoscope, CEA, Institut de Génomique, Evry, France; 5 Department of Plant Systems Biology, VIB, Ghent, Belgium; 6 Department of Plant Biotechnology and Bioinformatics, Ghent University, Ghent, Belgium; Universidad Miguel Hernandez, Spain

## Abstract

Among small photosynthetic eukaryotes that play a key role in oceanic food webs, picoplanktonic Mamiellophyceae such as *Bathycoccus*, *Micromonas*, and *Ostreococcus* are particularly important in coastal regions. By using a combination of cell sorting by flow cytometry, whole genome amplification (WGA), and 454 pyrosequencing, we obtained metagenomic data for two natural picophytoplankton populations from the coastal upwelling waters off central Chile. About 60% of the reads of each sample could be mapped to the genome of *Bathycoccus* strain from the Mediterranean Sea (RCC1105), representing a total of 9 Mbp (sample T142) and 13 Mbp (sample T149) of non-redundant *Bathycoccus* genome sequences. WGA did not amplify all regions uniformly, resulting in unequal coverage along a given chromosome and between chromosomes. The identity at the DNA level between the metagenomes and the cultured genome was very high (96.3% identical bases for the three larger chromosomes over a 360 kbp alignment). At least two to three different genotypes seemed to be present in each natural sample based on read mapping to *Bathycoccus* RCC1105 genome.

## Introduction

Small photosynthetic eukaryotes <3 µm in size [1] play an important role in marine ecosystems. In many oceanic regions, they may account for a large fraction of the biomass [2], [3] as well as of the primary production [4], [5]. Until now, only a small number of these organisms have been brought into culture [1], in particular members of the Chlorophyta (prasinophytes and Mamiellophyceae). Most of our knowledge about key groups and about diversity within these groups is based upon the genetic analysis of the 18S rRNA gene in marine samples. In many cases however, sequences obtained using classical approaches (universal primers applied to filtered samples) are dominated by heterotrophic eukaryote groups such as marine alveolates or stramenopiles. In order to focus on photosynthetic groups, three major approaches have been used: (1) cloning/sequencing of filtered samples of plastid genes such as 16S rRNA [6], [7] or *psbA* [8], coding for the D1 protein of the reaction center, (2) cloning/sequencing of the 18S rRNA gene of chlorophyll containing populations sorted by flow cytometry [9], [10], (3) direct detection of photosynthetic taxa by FISH (fluorescent *in situ* hybridization) probes or quantitative PCR [11], [12]. These different approaches, despite sometimes providing conflicting images of the small eukaryote community [9], have converged towards establishing that a few groups appear to dominate open-ocean, meso- and oligo-trophic waters: Prymnesiophyceae, Chrysophyceae, Pelagophyceae, and two clades of prasinophytes (VII and IX) [5], [9], [10], [13], [14]. In these waters, many of the dominant clades within these groups have very few or no cultured representatives, and no information is available on their morphology and physiology. In contrast, in temperate coastal as well as arctic pelagic waters, the small photosynthetic eukaryote community is dominated by Mamiellophyceae [11], [15], [16], a group of small green algae for which the three major genera *Bathycoccus*, *Micromonas*, and *Ostreococcus* are easily isolated in culture. These three genera have also been found to bloom sporadically in open ocean waters [17], [18]. The availability of cultures has allowed sequencing of their genomes [19], [20], providing key information on the genetic basis of their niche differentiation [21], [22] and fostering further analysis of metabolic pathways, mechanisms of genome evolution and life cycle of these organisms [23]–[25]. Obtaining genomic information from uncultivated populations would allow exploring their physiological adaptation and could provide clues for their isolation.

In the last decade, metagenomics approaches have been developed for marine microbial communities [26]. These approaches revealed the existence and ubiquity of processes such as photo-heterotrophy among bacteria [27]. However, until very recently metagenomics have not been applied to eukaryotic populations. The first reason is that filtered samples that are used in general for metagenomic studies are dominated by prokaryotic sequences [28]. Second, eukaryote genomes can be very large with many repeated genes, such that metagenomic data carry little information [29]. One approach, proposed a few years ago for prokaryotes [30], has been gaining popularity lately. It consists in the coupling flow cytometry sorting, which permits to obtain specific cellular groups based on their size and pigment content, with Whole Genome Amplification (WGA), which allows obtaining enough material for genome sequencing with next generation sequencing (NGS) technologies such as 454 pyrosequencing. This approach allowed, for example, reconstructing the genome of UCYN-A, a group of small nitrogen fixing cyanobacteria [31]. Recent work demonstrated that this strategy is applicable to small eukaryotes, both autotrophic [10], [32], [33] and heterotrophic [34]. This strategy allowed for example Yoon et al. [35] to retrieve genomic information on a group of uncultivated eukaryotes, the picobiliphytes [36].

In the present paper, we report metagenomic data from two natural picoplankton populations sampled in the Pacific upwelling off the Chile coast. The data are dominated by *Bathycoccus* sequences, which display high identity to the recently sequenced genome of a Mediterranean strain [37].

## Results

### Metagenomics of Sorted Samples

Photosynthetic picoeukaryotes from two samples collected in the nutrient-rich coastal upwelling waters off central Chile (samples T142 and T149 corresponding to 5 and 30 m depth, respectively, Table 1) were sorted by flow cytometry. As previously reported [32], [38], 18S rRNA gene clone libraries for these samples were dominated by members of the Mamiellophyceae, a novel class of small green algae previously classified within Prasinophyceae [39]. Among these, *Micromonas* and *Ostreococcus* dominated in sample T142, while *Bathycoccus* and *Micromonas* dominated in T149. In T149, sequences from Chrysophyceae and parasitic Syndiniales (Alveolata) were also recovered. The DNA from these samples was amplified by multiple displacement amplification (MDA). 18S rRNA gene clone libraries performed after MDA contained only *Bathycoccus* sequences, with the exception of one Acantharea sequence in T149 [32]. Each MDA amplified sample was sequenced on a 454 machine (half run) yielding about 670,000 reads, each with an average length ∼420 bp. Reads were assembled into contigs using either the default 454 assembler (Newbler) or the Geneious assembler after trimming to remove low quality ends (see Material and Methods). The former assembler yielded less contigs than the latter (Table 1) including fewer long contigs (>500 bp). Most of the following analyses have been done on the contigs generated by Geneious with the exception of the metabolic gene analyses (see below), which were performed early with Newbler contigs.

**Table 1:** Summary of metagenomic sequences and assemblies for eastern South Pacific picoeukaryote samples T142 and T149.

Sample Name	T142	T149
Cruise	BIOSOPE	BIOSOPE
Date	06/12/2004	08/12/2004
Station	UPW1	UPW3
Longitude	73° 22.177 W	73° 20.413 W
Latitude	33° 59.779 S	33° 51.630 S
Depth (m)	5	30
Population	Photosynthetic picoeukaryotes	Photosynthetic picoeukaryotes
Number of cells sorted	104 000	233 000
**Reads 454**		
Number	671 249	671 832
Total length (bp)	287 619 051	279 858 614
Mean length (bp)	429	417
Range (bp)	40-2015	40-2044
GC mean	46.7%	46.5%
**Contigs - Assembly Newbler**		
Number	17 633	28 262
Total length (bp)	16 984 438	24 845 872
Number > = 500 bp	9 010	13 213
Largest (bp)	35 494	43 276
**Contigs - Assembly Geneious**		
Number	23 187	34 839
Total length (bp)	22 907 873	34 947 661
Number > = 500 bp	15 074	22 219
Largest (bp)	28 395	36 675
Number of reads used for contigs	633 780	607 236

### Taxonomic Composition of the Metagenomic Sequences

In order to determine the taxonomic composition of the assemblage from which the metagenomic sequences originated, we began by searching for SSU rRNA gene within contigs (Table S1). Although the number of contigs harbouring SSU rRNA genes was slightly higher in sample T149 than T142, their phylogenetic composition was very similar. In both samples, we found nuclear 18S rRNA gene signature for *Bathycoccus* (99.9% identity) but not for other Mamiellophyceae. In contrast, we found plastid and mitochondrial 16S rRNA gene signatures for two other Mamiellophyceae, *Micromonas* and *Ostreococcus*. The only other eukaryotic signatures came from a Chrysophyceae (plastid) in T142 and from a Dictyochophyceae in T149. Although eukaryotes were targeted during flow cytometry sorting, we also found several 16S rRNA genes from bacteria in both samples. All bacterial signatures were affiliated to clades that are typical of the marine environment, in particular the alpha-proteobacteria *Candidatus* Pelagibacter and *Roseobacter*, as well as several Sargasso Sea clades (SAR) [40].

In a second step, we subjected both contigs and reads to a global BLASTN search against GenBank and analyzed the results with MEGAN, which provides, when possible, a taxonomic affiliation for each sequence using the Last Common Ancestor (LCA) algorithm [41]. The two samples T142 and T149 had very similar composition (Table S2). Slightly more than 30% of the reads were affiliated to eukaryotes with a small fraction (6% of total) corresponding to Mamiellophyceae, especially *Ostreococcus* (4% of total) and *Micromonas* (slightly more than 1% of total). It should be noted that while we clearly identified *Bathycoccus* as a key eukaryote in both samples based on rRNA analysis, the number of reads attributed to this genus by BLASTN analysis was small because the genome of *Bathycoccus* was not available in public databases at the time of this analysis. The contribution of other eukaryotic groups expected to be encountered within picoeukaryotes [1] such as stramenopiles, haptophytes, or alveolates was small, which is in part explained by the low number of sequenced genomes available for these groups. About 8% of the reads were affiliated to bacteria especially Proteobacteria such as *Candidatus* Pelagibacter (SAR11), confirming the results from the rRNA gene analysis. Very few reads were attributed to Archaea or viruses. The majority of reads (∼60%) could not be affiliated to any taxon either because they had no hits in GenBank or because the LCA of their hits was unresolved (for example, if the best hits consist of a mixture of eukaryotic and bacterial sequences, the LCA will be ‘cellular organism’). MEGAN analysis of Geneious contigs provided similar results, with an increased fraction of contigs, when compared to reads, attributed to bacteria (18%), and therefore fewer contigs with unknown affiliation (50% *vs.* 60% for reads).

Key problems with the public databases are the over-representation of irrelevant sequences and the absence of relevant sequences. Analysis of the SSU rRNA gene both in clone libraries (see above) and in contigs (Table S1) suggested that *Bathycoccus* was the dominant eukaryote in both samples. At the time of this analysis, we gained access to the genome sequence of a *Bathycoccus prasinos* strain (RCC1105) isolated from the Mediterranean Sea [37], which was not yet publicly available then, but has been deposited to Genbank since (see Material and Methods). In order to estimate the fractions of contigs that could belong to *Bathycoccus*, we constructed a protein database including proteins from nuclear genomes of *Bathycoccus* RCC1105 and unicellular eukaryotes (Mamiellophyceae, diatoms, haptophytes, pelagophytes, fungi), mitochondria and plastid genomes from selected eukaryotes, genomes from typical marine archaea and bacteria such as that of the bacterium *Candidatus* Pelagibacter, unicellular cyanobacteria or *Thermococcus*, as well as a few eukaryotic virus genomes. Contigs were searched with BLASTX against this database and results were filtered using an algorithm specifically designed to extract Mamiellophyceae sequences using fine-tuned identity and coverage thresholds for each orthologous protein sequence (see Material and Methods). Again, results were similar for both samples (Table S3). On average for the two samples, 31.5% of the contigs could be attributed to *Bathycoccus*, 0.45% to *Micromonas* and 0.36% to *Ostreococcus.* This corresponded to 69.8, 0.10, and 0.12% of the reads, respectively, indicating that *Bathycoccus* contigs had a higher coverage than contigs belonging to other organisms. About 4.7% of contigs (5.0% of reads) could be assigned to Mamiellophyceae without a clear relationship to any of the three genera. Very few contigs could be attributed to the other eukaryotic genomes available. Among prokaryotes, only *Candidatus* Pelagibacter strain HTCC1062 genome recruited a significant number of contigs, in agreement with the MEGAN and rRNA gene analyses reported above. A few contigs (5 for T142 and 17 for T149) could be assigned to available large double stranded Mamiellophyceae viruses [42].

The predicted protein sequences from T142 and T149 contigs attributed to *Bathycoccus* had an average amino-acid identity of 96.0 and 95.9%, respectively, with those from the *Bathycoccus* sequenced genome. This amino-acid identity is an overestimate because our taxonomic affiliation only retains genes with greater than 80% amino-acid identity (see Materials and Methods). The detailed assignment of contigs to individual *Bathycoccus* chromosomes (Table S4) demonstrated some differences between the coverage of each chromosome. Specific chromosomes such as the outlier chromosomes 14 and 19 [37] or the chloroplast genome had a lower coverage.

### Assembly of T142 and T149 Reads Against the *Bathycoccus* RCC1105 Genome

**Figure 1:**
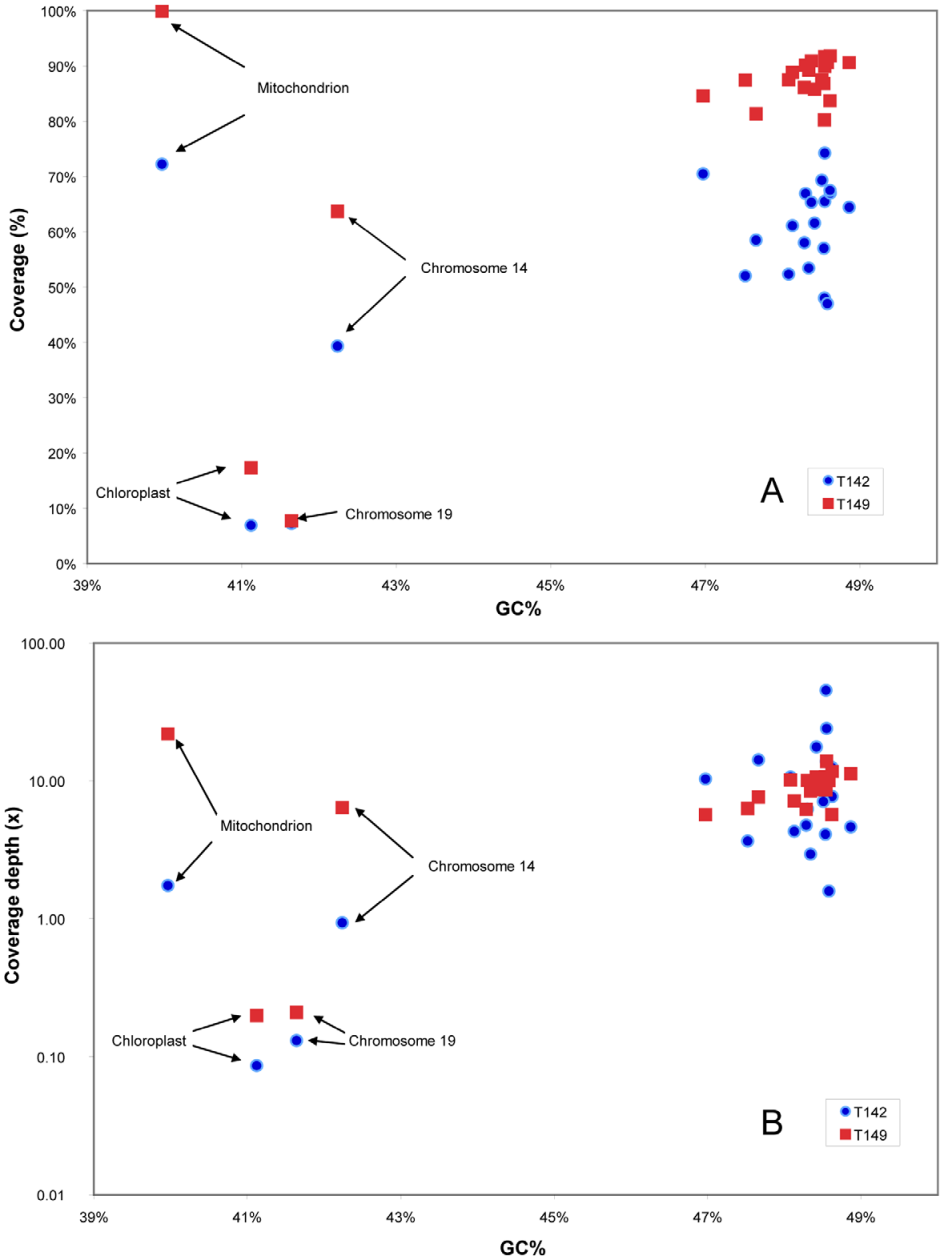
Assembly of metagenomic reads obtained from flow cytometry sorted picoeukaryote samples T142 and T149 from the Chile upwelling to *Bathycoccus prasinos* RCC1105 genome. (A) Relationship between average coverage fraction (expressed as % of the length of the chromosome covered by at least one read) and GC content for samples T142 and T149 for the 21 draft nuclear chromosomes of *B. prasinos* RCC1105 as well as the mitochondrion and plastid genomes. (B) Idem for average coverage depth (number of reads at each position).

**Figure 2:**
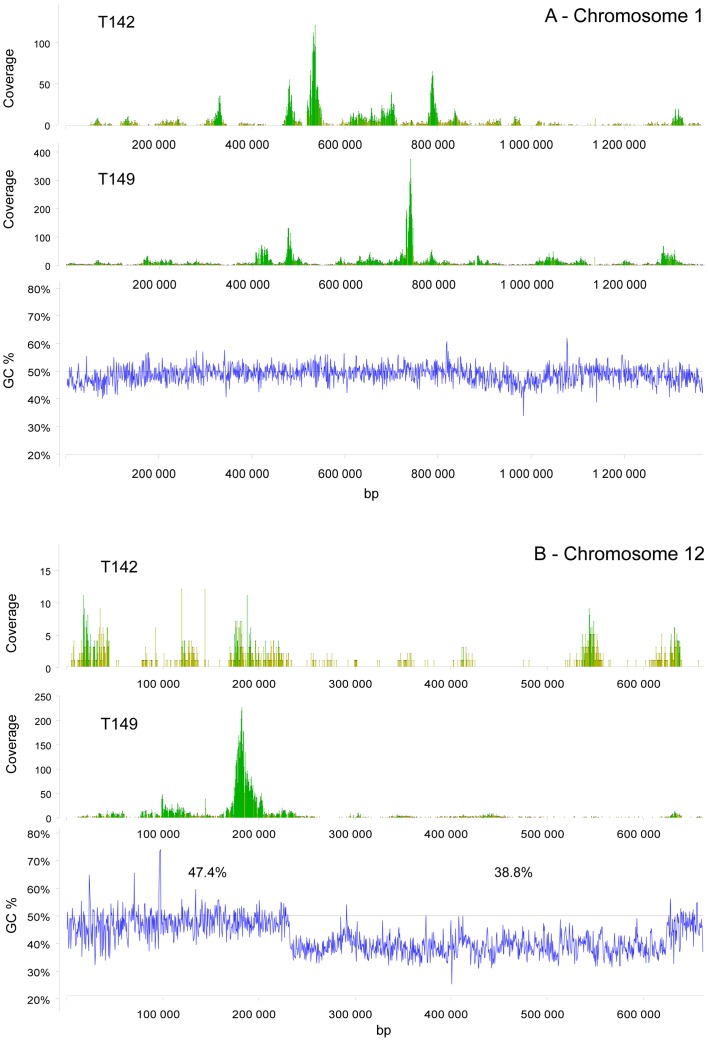
GC content and coverage depth by reads from the eastern South Pacific picoeukaryote samples T142 and T149 of individual chromosomes of *B. prasinos* RCC1105. (A) Chromosome 1. (B) Chromosome 14.

Since BLASTX analysis revealed that a large fraction of the contigs could be recruited to *Bathycoccus* RCC1105 genome, a direct assembly of reads was performed against the sequenced *Bathycoccus* genome (Table 2). Direct assembly was coherent with contigs assignment through BLASTX (Table S4). There was a good correlation between the numbers of reads recruited by both approaches with a slightly higher number of reads (about 3%) for the latter (Figure S1). This was expected because direct assembly relies on nucleotide identities and is therefore more stringent. Direct assembly provides detailed information on the genome coverage. On average, while coverage depth (i.e., the average number of reads covering a given base from the reference chromosome) was around 10× and similar between both samples, the coverage fraction (i.e., the fraction of the reference genome covered by at least one read) was higher in T149 (88%) than in T142 (62%). Both coverage depth and coverage fraction varied widely among chromosomes (Table 2), the two indices being somehow related since chromosomes with a higher coverage fraction had also generally a larger coverage depth. While in general individual chromosomes followed the general trend with sample T149 having a better coverage than T142, for some chromosomes such as 2 or 7, average coverage depth was higher for T142. In fact, coverage fraction and depth appeared much more variable for T142 than for T149 (Figure 1A). A weak relationship was observed between nuclear chromosome coverage and GC content (Figure 1A; Spearman correlation, T142: ρ = 0.40 p = 0.04; T149 ρ = 0.57, p = 0.004). In particular, the two *Bathycoccus* outlier chromosomes 14 and 19 and the chloroplast genome that have lower GC content [37] had low coverage fraction and low coverage depth (Figure 1). Surprisingly, the mitochondrial genome had a very good coverage despite its low GC content. When looking at the detail of coverage depth along each chromosome, the situation appeared much more complex (Figure 2). Coverage appeared very unequal and varied between the two samples. Some regions, for example the one located between positions 765 and 780 kbp on chromosome 1 (Figure 2A), were well-covered in both samples but most other high coverage regions were present in only one of the samples. In general, no correlation was detected between coverage and GC content along a given chromosome (data not shown). However, for the big outlier chromosome 14 which can be divided into regions of different base composition, two (at the beginning and very end) with the standard GC content at 47% and one in the middle with a lower GC content at 39%, the latter region had a low coverage (Figure 2B).

**Table 2:** Assignment of reads from samples T142 and T149 to individual chromosomes of *B. prasinos* RCC1105 using Geneious Assembler (see Materials and Methods for details).

				T142					T149				
Chromosome final	Chromosome draft	Length	GC%	Reads	Coverage	Coverage	Coverage Depth	Identical sites	Reads	Coverage	Coverage	Coverage Depth	Identical sites
		bp	%	#	bp	%	x	%	#	bp	%	x	%
chromosome_1	Bathy_chrom000	1 352 574	48.87%	17 507	887 310	64.40%	4.61	95.50%	44 115	1 260 242	90.60%	11.26	90.50%
chromosome_2	Bathy_chrom001	1 122 692	48.55%	76 131	763 755	65.50%	23.96	91.70%	45 448	1 045 505	89.90%	13.84	88.90%
chromosome_3	Bathy_chrom002	1 089 374	48.62%	23 634	747 266	67.00%	7.68	94.50%	36 961	1 032 050	91.80%	11.66	90.00%
chromosome_4	Bathy_chrom003	1 037 991	48.29%	14 217	613 146	58.00%	4.76	95.70%	19 099	914 632	86.10%	6.22	91.90%
chromosome_5a	Bathy_chrom012	550 167	48.34%	4 559	299 250	53.40%	2.95	96.50%	13 535	504 509	89.20%	8.44	90.90%
chromosome_5b	Bathy_chrom016	467 783	48.58%	2 152	222 904	47.00%	1.58	97.20%	13 712	437 480	90.60%	10.02	90.00%
chromosome_6	Bathy_chrom004	989 707	48.30%	17 733	677 434	66.90%	6.25	94.30%	29 292	915 584	90.10%	10.06	90.60%
chromosome_7	Bathy_chrom005	955 054	48.42%	49 892	608 532	61.60%	17.54	93.30%	30 116	845 293	85.80%	10.63	90.30%
chromosome_8	Bathy_chrom006	937 610	48.54%	10 892	545 919	57.00%	4.09	95.70%	29 489	841 544	86.80%	10.70	89.90%
chromosome_9	Bathy_chrom007	895 347	48.51%	17 995	636 008	69.30%	7.07	93.80%	23 672	806 272	87.50%	9.05	90.70%
chromosome_10	Bathy_chrom008	794 148	48.38%	20 350	533 074	65.30%	9.03	93.70%	19 996	743 420	90.90%	8.66	90.20%
chromosome_11	Bathy_chrom009	741 502	48.62%	26 119	516 527	67.50%	12.50	92.40%	12 368	634 120	83.70%	5.70	92.50%
chromosome_12a	Bathy_chrom019	201 229	47.52%	2 066	106 614	52.00%	3.66	96.30%	3 707	180 588	87.40%	6.31	91.40%
chromosome_12b	Bathy_chrom014	511 334	48.55%	19 985	391 273	74.20%	13.84	92.10%	15 530	483 876	91.70%	10.45	89.30%
chromosome_13	Bathy_chrom010	706 576	48.54%	90 889	363 302	48.00%	45.29	91.30%	17 849	584 838	80.20%	8.54	91.00%
chromosome_14	Bathy_chrom011-50	662 304	42.24%	1 873	264 505	39.30%	0.93	97.70%	12 822	433 085	63.70%	6.41	93.90%
chromosome_15	Bathy_chrom013	519 535	48.13%	6 320	324 598	61.10%	4.30	94.80%	10 865	473 213	88.80%	7.14	91.60%
chromosome_16	Bathy_chrom015	481 036	48.08%	14 765	268 376	52.30%	10.67	94.00%	14 526	445 984	87.50%	10.16	89.90%
chromosome_17	Bathy_chrom017-28	465 570	47.66%	18 609	283 891	58.50%	14.21	91.80%	10 433	390 682	81.30%	7.63	90.60%
chromosome_18	Bathy_chrom018	310 170	46.97%	9 003	226 193	70.50%	10.30	91.20%	5 203	269 409	84.60%	5.67	91.00%
chromosome_19	Bathy_chrom020	146 238	41.65%	73	10 779	7.20%	0.13	99.30%	129	11 504	7.70%	0.21	99.10%
Chloroplast	Bathy_chrom021	54 761	41.12%	21	5 029	6.90%	0.09	99.70%	46	12 675	17.30%	0.20	99.40%
Mitochondrion	Bathy_chrom024	42 168	39.97%	261	30 825	72.20%	1.74	97.00%	3 470	43 650	99.90%	21.90	91.70%
													
	**Total or mean**	15 034 870		445 046	9 326 510	62.03%	10.38		412 383	13 310 155	88.53%	9.34	
	% of reads			66.3%					61.4%				

The numeration of chromosomes is provided both for the draft version of the genome (used in this work) and for the final version of the genome [37].

### Genetic Diversity Among *Bathycoccus* Metagenomes and Cultured Genome

We analyzed the degree of similarity between the *Bathycoccus* metagenomes and the genome of *Bathycoccus* RCC1105 using two different approaches.

First, we selected 153 genes from *Bathycoccus* RCC1105 belonging to important metabolic groups (photosynthesis, pigment and vitamin synthesis, cell cycle). These genes were searched by BLASTN among contigs assembled with Newbler for T142 and T149 samples (Table S5). We identified 100 (65.4%) and 137 (89.5%) homologous genes, for T142 and T149 respectively. These percentages are coherent with the average coverage obtained by direct assembly (Table 2). Nucleic acid identities averaged 98.3% and 98.4% for T142 and T149, respectively, and were similar between the two samples for each gene group. For some gene groups, in particular genes involved in vitamin synthesis, such as *bioB* (biotin synthase), we observed some amino acid deletions, often within proteins containing stretches with repeated amino acids, in particular serine.

In order to compare more globally the metagenomes from samples T142 and T149 to the *Bathycoccus* genome, we aligned the RCC1105 genome sequence and the T142 and T149 consensus sequences for the three largest chromosomes (1 to 3). We removed all regions that had a coverage depth below 10× for both metagenomes and obtained three alignments varying between 78 and 153 kbp (Table 3). The percentage of identical nucleotides over all positions of three genomes varied between 95.8% and 96.7%. However for non-coding regions, this identity was lower between 87.2 and 89.5%. The percentage of identical nucleotides was in general higher between T142 and T149 than between RCC1105 and any of the two metagenomes (Table 3).

**Table 3:** Similarity between *B. prasinos* RCC1105 genome and T142 and T149 assemblies for the three larger chromosomes.

	Length alignment (bp)			% Identical sites			% Identical sites (CDS + non CDS)		
	CDS + non CDS	CDS	non CDS	CDS + non CDS	CDS	non CDS	Bathy vs T142	Bathy vs T149	T142 vs T149
chromosome_1	78 022	70 644	7 376	96.7%	97.5%	89.5%	97.8%	97.3%	98.3%
chromosome_2	158 544	142 659	19 681	95.8%	97.0%	87.2%	97.0%	96.6%	97.4%
chromosome_3	123 912	115 661	12 823	96.7%	97.4%	89.5%	97.8%	97.4%	97.8%
**Total/Average**	360 478	329 964	39 880	96.3%	97.2%	88.5%	97.4%	97.0%	97.7%

Only regions with coverage in excess 10× for both samples were considered. Total genome and non-CDS regions were analyzed separately (see Material and Methods for details).

**Figure 3:**
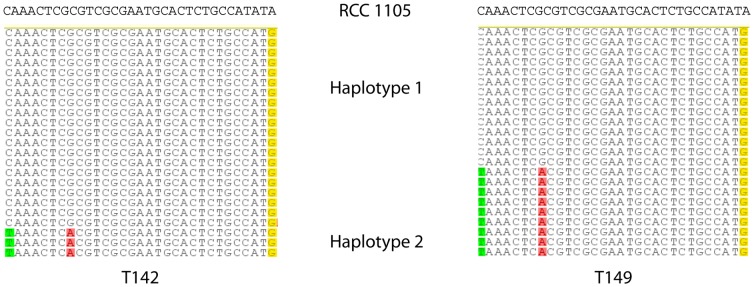
Genotypes observed for *B. prasinos* RCC1105 gene Bathy02g01050 (pigment synthesis protein, BOGAS annotation code) for samples T142 (left) and T149 (right). At least two different sequences appear to be present in both samples, differing by one and three positions, respectively, from the reference *B.prasinos* RCC 1105 sequence. Only 23 representative reads are shown for each sample although more reads covered this region (see Table S6).

Detailed analysis of the reads mapped to specific regions of *Bathycoccus* single-copy genes that have a good coverage allowed estimating the number of different *Bathycoccus* genotypes present in each sample. For example, for gene Bathy02g01050 (gene code according to BOGAS web site – see Materials and Methods) involved in pigment synthesis (Figure 3), sample T142 presented two major sequences accounting for 85 and 15% of the reads, respectively (Table S6). In the same region of sample T149, the same two genotypes were present but in different proportion, 59 and 41%, respectively. Examination of a few regions for genes that had a good coverage revealed that the number of different sequences varied from 1 to 3 per samples (Table S6). In some cases, the same sequences were present in both samples, while in other cases unique sequences were present in each sample (Table S6).

A thorough analysis was performed on the rRNA operon. The SSU rRNA gene in the RCC 1105 genome contains a 433 bp intron (Figure S2A), which is lacking in several other *Bathycoccus* strains [37]. The two Geneious contigs from samples T142 and T149 (contigs T142_109 and T149_486) containing the rRNA operon were also lacking this intron (Figure S2B). However, the examination of the individual reads revealed that sample T149 contained one read (Figure S3A) with a sequence nearly identical to the beginning of the intron sequence, suggesting the presence of two genotypes in this sample, one containing the intron, the other lacking it. Contig T142_109 appeared to contain a 468 bp intron at the end of the LSU rRNA gene (Figure S2B). Reads with this intron signature were dominating in sample T142 (10 out of 15, Figure S3B). However, the signature of the LSU intron was also found in one read from sample T149 (Figure S3C). Besides these features, genotype variation was detected at 4 positions within the 5646 bp rRNA operon (Table S7), one within the SSU rRNA gene and three in the ITS2 region.

## Discussion

Until recently, most metagenomic studies have focused on prokaryotes. However in the last year, three papers have reported application of metagenomics to marine eukaryotes [10], [33], [35]. These studies relied on the physical separation of eukaryotic cells from the dominant bacteria by flow cytometry sorting, whole genome amplification, and next generation sequencing. We used the same strategy on two samples from the coastal upwelling off Chile obtained during the 2004 BIOSOPE cruise from which photosynthetic picoeukaryotes were sorted. Following Cuvelier et al. [10] and Monier et al. [33], but in contrast to Yoon et al. [35], we sorted large cell populations and not single cells. Therefore the DNA, although obtained from cells that were homogeneous in terms of size and pigment content, corresponded to an assemblage of species and genotypes as demonstrated by previous analyses of rRNA genes on the same samples [9], [38].

The DNA amplified by MDA from sorted cells [32] proved to be suitable for 454 sequencing, giving a number of reads adding up to more than 550 Mbp for the two samples (Table 1). Our initial assumption was that genotypic diversity would be low in our samples, that genomes of the targeted organisms will be rather small (typically 20 Mbp), and that we would be able to reconstruct long contigs. However, the use of two different *de novo* assembly programs (Newbler and Geneious) yielded maximum contig lengths around 40 kbp (Table 1), quite similar to those obtained by Cuvelier et al. [10], shorter than those obtained by Monier et al. [33], but longer than those reported by Yoon et al. [35].

Assigning reads and contigs to sequenced genomes is much less straightforward for eukaryotes than for prokaryotes because of the paucity of unicellular eukaryote genomes available. This is particularly true for marine photosynthetic microalgae, for which less than 15 genomes have been sequenced. In a first step, we used SSU rRNA genes for which a very large database is available [43] in order to determine the phylogenetic composition of the sequenced community. This indicated that Mamiellophyceae, in particular *Bathycoccus*, were present in the amplified sample as previously shown by the construction of 18S rRNA gene clone libraries from the same samples [32]. The three Mamiellophyceae genera *Bathycoccus*, *Micromonas*, and *Ostreococcus* have been shown to be important in coastal areas [11]. More specifically, the presence of *Bathycoccus* in the upwelling zone is coherent with the recent observation that this genus makes on average 47% of photosynthetic picoeukaryote carbon in this region with maxima up to 78% in autumn, at the same period of the year than our samples [44]. The two other eukaryote SSU rRNA signatures (Dictyochophyceae, Chrysophyceae) correspond to groups often present within picoeukaryote communities from the eastern South Pacific [9]. The presence of marine bacteria SSU rRNA signatures, in particular of *Candidatus* Pelagibacter, could be explained either by the fact that bacteria may be sorted in the same drop than photosynthetic cells (we did not fluorescently label bacteria to exclude them), or by the fact that some of the photosynthetic picoeukaryotes maybe mixotrophs and may have ingested bacteria [45], [46].

The use of BLASTN to assign either reads or contigs (Table S2) against publicly available sequences yielded a very large number of “unknown” sequences (around 60% for reads and 50% for contigs). This analysis confirmed however the dominance of eukaryotic compared to prokaryotic sequences, as expected, and among the former the importance of the green lineage. Still, very few sequences could be assigned at the genus level. The construction of a data set including the *Bathycoccus* genome [37], and the use of a BLASTX-based algorithm, revealed that 70% of the reads assembled into contigs could be assigned to *Bathycoccus* (Table S3). This illustrates the difficulty to assign eukaryotic metagenomic data in the absence of closely related reference genomes. Even the presence in the public databases of genomes for representatives of the closely related genera *Micromonas* and *Ostreococcus* did not allow recruitment of *Bathycoccus*-related sequences.

The prevalence of *Bathycoccus* sequences allowed performing a direct assembly of the reads from the two Pacific samples to the genome sequence of the Mediterranean strain RCC1105 (Table 2). This confirmed that a large fraction of the reads could be assigned to *Bathycoccus* and there was an excellent correlation between the number of reads that could be assigned directly to *Bathycoccus* chromosomes and those assigned after contig assembly and BLASTX analysis of the contigs (Figure S1). Direct assembly revealed however that coverage was extremely uneven across and between chromosomes (Figure 2). For example, on Chromosome 2, some bases where covered with a depth in excess of x1,000. Such uneven coverage is very likely to be due to intrinsic characteristics of the MDA process and has been clearly illustrated on *Prochlorococcus* [47]. By amplifying seven single cells and sequencing the amplified DNA with two different technologies (454 and Illumina), the authors observed very unequal coverage. The coverage was not depending on the sequencing technology for a given cell, but differed randomly between cells. In our case, we did not find any relationship between coverage variation along a given chromosome and GC content, a factor often invoked to explain these variations, probably because GC content variation within most chromosomes is low. One exception is the big outlier chromosome 14, which is made of three parts, the middle one having a 10% lower GC content and exhibiting a very low coverage (Figure 2). This low coverage could also be due to a higher genetic variation in this region exemplified by the fact that this chromosome lacks co-linearity with the other Mamiellophyceae outlier chromosomes [37]. When considering average coverage for each chromosome and average GC content, there appears to be a clear relation both in terms of coverage depth and coverage fraction (Figure 1). One interesting exception is the mitochondrion which had a good coverage despite its low GC content. Previous analyses of picoeukaryotic sequences from the Sargasso Sea metagenome revealed a higher prevalence of chloroplast and mitochondrial sequences relative to nuclear sequences in *Ostreococcus*, suggesting that both organelle genomes are present in multiple copies per cell [48]. This higher coverage could also be due to genome conformation, since it has been reported that circular genomes are better amplified by MDA than linear ones [49]. However genome conformation and/or multiple genome copy number should also have induced a good coverage of the chloroplast genome, which is not the case in our dataset.

The *Bathycoccus* Mediterranean culture genome and the two Chile metagenomes that originate from opposite sides of the Earth appear very closely related, with an average nucleotide identity up to 98% at the individual gene level (Table S5). A more extensive comparison of the three genomes made by aligning the cultured genomes and the assembly consensus for the first three chromosomes revealed an average of 96.3% of identical sites in regions with high coverage (in excess of 10× for both samples, Table 3). Non-coding regions displayed a lower number of identical sites (88.5%, Table 3) than the coding regions, as expected due to lower constraints in the non-coding regions [50]. There were no significant differences between the three chromosomes examined. Samples T142 and T149 shared more identical sites between them than either of them with RCC1105. However, differences remained small. Conservation between these three genomes is much higher than, for example, between the genomes of *O. tauri* and *O. ‘lucimarinus’*, for which average amino-acid identity is only 88% when using our BLASTX-based algorithm to affiliate *O. ‘lucimarinus’* sequences (random 1,000 bp sequences) to *O. tauri* genome taken as a reference. In fact, it is possible that all three genomes correspond to the same biological species, since an analysis of the full rRNA operon revealed nearly identical sequences in the ITS2 region (Figure S2, Table S7) and identical sequences in the ITS2 have been shown to correspond in general to reproductive compatibility [51]. In our data, genotype variability in the ITS2 region occurs outside of helix 3 (Table S7), therefore outside the region where differences are clearly associated with reproductive incompatibility in many species [51]. However, several studies on multiple genes or complete genomes have shown that sequence identity is just one of the hallmarks of reproductive isolation and speciation, because chromosomal translocation or duplication may hamper species compatibility whilst most sequences remain identical [52].

Despite the high level of sequence identity between these three genomes, several genotypes appear to be present within each of the Chile upwelling populations. This is clearly seen in the detailed analysis of the rRNA operon. The SSU rRNA intron that is present in the cultured RCC1105 genome was absent from the two contig consensus sequences obtained for the upwelling samples T142 and T149 (Figure S2). However one read from sample T149 matched nearly perfectly the RCC 1105 intron while all other T149 reads from this region presented no intron signature. This suggests the presence of two genotypes in this sample, one dominant not possessing the intron, the other containing it. Another putative intron was found near the end of the LSU rRNA gene. This intron was present in a fraction of the reads for sample T142. The other T142 reads matched perfectly RCC 1105, which lacks this intron. In sample T149, one read out of 16 also matched the putative LSU intron (Figure S3). This again suggests the existence of at least two genotypes in these two samples, one with the LSU intron, one without. Analysis of specific genome regions that have a good coverage for both samples (Figure 3, Table S6) provided further evidence for the existence of two to three genotypes per sample. Individual genotypes were usually present in both samples, but, in some cases, genotypes from the two samples were completely different (Table S6).

Monier et al. [33] have very recently reported the construction of a *Bathycoccus* metagenome (GenBank accession AFUW01000001:AFUW01000185) from flow cytometry sorted cells collected at the deep chlorophyll maximum (DCM) in the tropical Atlantic. We compared, using our custom BLASTX-based algorithm (see Materials and Methods), the DCM metagenome and the RCC1105 genome. Amino acid identity with the reference *Bathycoccus* RCC1105 genome was much lower (84.4%) than for our metagenomes (96.0 and 95.9%, for T142 and T149, respectively), despite the fact that the SSU rRNA sequence of the DCM metagenome is 100% identical to that of RCC1105 (but it lacks the intron). This raises the intriguing possibility that the *Bathycoccus* genus could contain different ecotypes, some adapted to coastal waters, the others to pelagic/deep waters, in a manner similar to its sister genera *Ostreococcus* [53] and *Micromonas* [54]. More detailed comparisons, clearly outside the scope of the present paper, will be needed to confirm this hypothesis.

This work illustrates the power of coupling flow cytometry sorting to target specific populations followed by NGS to obtain sequence data on photosynthetic microbes as shown in recent papers [10], [55]. The availability of a sequenced genome for the dominant organism in these samples proved to be crucial for the data analysis. Our data and that of Monier et al. [33] suggest that the ubiquitous *B. prasinos* may contain several ecotypes as well as different genotypes within natural coastal populations. The application of this approach, provided that higher and more uniform coverage can be obtained, may bring in the future unique information on uncultured small photosynthetic eukaryotes that appear to dominate in the more oligotrophic oceanic regions [38].

## Materials and Methods

### Sampling

Sampling was performed in December 2004 during the oceanographic cruise BIOSOPE [56] that sailed a transect through the eastern South Pacific Ocean on board the research vessel L’Atalante. No specific permits were required for the described field studies. The location (Pacific Ocean) is not privately-owned or protected in any way. The field studies did not involve endangered or protected species. Seawater was collected using Niskin bottles mounted on a CTD frame at two very closely located stations in the Chile upwelling (UPW1 and UPW3) at 5 and 30 m, respectively (Table 1). Samples were concentrated by tangential flow filtration using a 100 000 MWCO (Regenerated Cellulose- RC ref VF20C4) Vivaflow 200 cassette [38].

### Flow Cytometry Analysis and Sorting

Concentrated samples were analyzed on board using a FACSAria flow cytometer (Becton Dickinson, San Jose, CA, USA) equipped with a laser emitting at 488 nm and the normal filter setup. The signal was triggered on the red fluorescence from chlorophyll. Photosynthetic picoeukaryotes were discriminated based on side scatter, as well as orange and red fluorescence, and sorted in “purity” mode. Cells were collected into Eppendorf tubes and, after a quick centrifugation, the volume of sorted samples was adjusted to 250 µL by adding filtered seawater. Samples were deep frozen in liquid nitrogen.

### DNA Extraction and Amplification

DNA from the sorted pico-eukaryote population was extracted using DNeasy blood and tissue kit (Qiagen), as recommended by the manufacturer. Multiple displacement amplification (MDA) was performed using the REPLI-g Mini kit (Qiagen) following the manufacturer’s protocol with modified buffers as described previously [32]. Briefly, one µL of DNA (corresponding to 3–5 ng of DNA) was used as the template in the MDA reaction. Reactions were carried out in 50 µL volumes. Reaction buffer (29 µL), water (9.5 µL), and 1 µL of Phi29 DNA polymerase were added to 10.5 µL of template (corresponding to 1 µL of DNA, 2.5 µL of phosphate-buffered saline, 3.5 µL of an alkaline solution and 3.5 µL of neutralization buffer) and incubated at 30°C for 16 h. A final incubation at 65°C for 5 min inactivated the Phi29 DNA polymerase. Twelve separate reactions were performed and then pooled together for both T142 and T149 samples in order to reach the 10 µg of DNA required for 454 sequencing. The amplified products were then purified and concentrated using a Microcon YM- 100 column (Millipore, Molsheim, France). After WGA, 5 µL of the amplified product was run on an agarose gel (1%) in order to estimate amplification efficiency. DNA was also quantified in the final reaction volume (before and after purification/concentration) with Quanti-iTTM PicoGreen dsDNA (Invitrogen, Carlsbad, CA), as described previously [32].

### Sequencing

About 10 µg of DNA amplified were fragmented by nebulisation. Fragments between 500 and 800 bp were selected and purified by AMPure (Beckman Coulter Genomics). Libraries were prepared following 454 protocol (GS FLX Titanium Library Preparation Kit, Roche Diagnostic, USA). Libraries were quantified and libraries profiles were evaluated using a 2100 Bioanalyzer (RNA 6000 PicoLabchip kit, Agilent Technologies, USA). Each library was sequenced using 1/2 Pico Titer Plate on 454 GSFlx instrument with Titanium chemistry (Roche Diagnostic, USA). About 671,000 reads were obtained for each sample.

### Genome Assembly – *de novo*

Two types of assemblies were performed from the raw reads. First the native 454 assembler Newbler was used with the defaults Assembly parameters (overlap Seed Step  = 12; overlap Seed Length  = 16; overlap Min Seed Count  = 1; overlap Seed Hit Limit  = 10000; overlap Hit Position Limit  = 200; overlap Min Match Length  = 40; overlap Min Match Identity  = 90; overlap Match Ident Score  = 2; overlap Match Diff Score  =  −3; overlap Match Unique Thresh  = 12; map Min Contig Depth  = 1; all Contig Thresh  = 100; auto Trimming  =  true; true Pair Distance Thresh  = 5000). Summary of assembly results are provided in Table 1. However one problem with the Newbler assembly is that the same read may appear into different contigs. Therefore we performed a second assembly using the native Geneious Assembler ([57], available at http://www.geneious.com/). First, 454 reads were trimmed at both ends using a probability threshold of p = 0.01 and no ambiguity. Trimmed reads were assembled (Table 1) using the default Medium Sensitivity (Allow Gaps  =  true; Word length  = 14; Index word length  = 12; Ignore words repeated more than 200 times; Maximum mismatches per reads  = 15%; Maximum ambiguity  = 4; Maximum gap size  = 2).

### Genome Assembly – *Bathycoccus* Genome

Trimmed 454 reads from samples T142 and T149 were assembled against the genome of *Bathycoccus* (downloaded with annotations for CDS [coding sequences], UTR [untranslated regions], introns and exons from the University of Ghent BOGAS web site http://bioinformatics.psb.ugent.be/webtools/bogas/overview/Bathy) using Geneious with the default “Medium Sensitivity” (see previous paragraph). Since analysis was performed on an initial draft version of the genome, the correspondence between the initial and the final naming of the chromosomes has been provided in the different tables (e.g. Table 2). In the text, we used the final chromosome numbering [37]. In order to test whether the large numbers of reads that could be assembled to *Bathycoccus* genome were not an artefact, assembly of T142 reads was also performed against the genome of *O. ‘lucimarinus’* [21]. Only 1,640 reads could be assembled (Table S8) vs. 445,046 when *Bathycoccus* is used as a reference (Table 2).

### Contigs Containing SSU rRNA Genes

We searched for rRNA SSU genes within Geneious contigs by performing a BLASTN analysis against the annotated Silva rRNA database (version 104) [43]. Hits longer than 200 bp were subjected to a second BLAST search against the NR GenBank database to confirm the presence of a rRNA gene.

### BLASTN and MEGAN Analysis of Reads and Contigs

We performed a BLASTN search of the raw reads and Geneious contigs against a subset of nr GenBank database, after removing certain taxa and sequence sets that yielded non-relevant hits (Invertebrates: gb inv; mammals: gb mam; primates: gb pri; rodents: gb rod; vertebrates: gb ver; sequence tagged sites: gb sts; genome survey: gss; high throughput genomic: gb htg; synthetic: gb syn). Results from the BLASTN search were analyzed with MEGAN 4.0 [41] with the standard parameters (min score  = 35, top-percent  = 10%, min support  = 5) in order to provide a taxonomic affiliation for each read and contig.

### BLASTX Analysis of Contigs

We performed a stringent BLASTX-based two-step approach. First, we used a reference protein database (Table S9) containing gene annotations from 11 genomes of photosynthetic unicellular eukaryotes (targeted by this study), 2 non-photosynthetic eukaryotes, 7 marine bacteria and 8 viruses of planktonic eukaryotes. In particular, we included the genome of *Bathycoccus*, which was not yet publicly available at the time of this analysis [37]. Translation from each contig was compared against the protein database using BLASTX [58]. The local alignments provided by BLAST were merged into one non-redundant global alignment for each contig and its Best BLAST Hit (BBH), checking which position of the contig was covered in a local alignment with its BBH.

We pre-computed the identity (frequency of matching sites over the alignment), *I*, and alignment length, *L*, (number of sites present in the global alignment) between all Mamiellophyceae genes and between Prasinovirus genes. For each protein having an orthologous gene, we identified minimum identity percent *I_min_* and coverage length *L_min_* within the genus or within the class. For specific genes, i.e. genes present in only one genome, we set up a minimum of 80% identity over 50 amino acids.

For example, the *O. ‘lucimarinus’* EF1-alpha gene has a 76% amino-acid identity over a 476 amino-acids alignment within the genus *Ostreococcus* and a 48% identity over a 434 amino-acid alignment within Mamiellophyceae. We would thus assign a contig to the genus *Ostreococcus* if it matched *O. ‘lucimarinus’* EF1-alpha gene with an identity greater than 76% and an alignment length greater than the observed alignment length. If either the identity or the alignment lengths were smaller than these thresholds, but still larger than the thresholds within Mamiellophyceae, we would assign this contig to Mamiellophyceae. If a contig did not match any gene with pre-computed thresholds, it was assigned to the taxonomic affiliation of its BBH using a minimum identity threshold of 80% over 50 amino-acids. The corresponding C codes are available upon request from ET (Toulza, in prep).

The sensitivity of our approach was tested by estimating the taxonomic affiliation of random samples of the nuclear genome of *O. ‘lucimarinus’* using a Mamiellophyceae protein database and a pre-computed identity and threshold matrix without this species. As expected, our approach significantly lowered the rate of false positive taxonomic affiliation from 25.1% to 2.1% for 500 bp sequences and decreases with sequence length (Figure S4).

### Gene Specific Similarity between the Three Genomes

For specific groups of genes that are important for phytoplankton metabolism (photosynthesis, vitamin and pigment synthesis, cell cycle) we examined the similarity between the genome of the cultured *Bathycoccus* strain and the metagenomes T142 and T149. A list of 153 genes was defined from the *Bathycoccus* RCC1105 genome by using the sequences of orthologous genes from the previously published *Ostreococcus* and *Micromonas* genomes, as baits for BLASTN search against the *Bathycoccus* genome. After proper identification, each *Bathycoccus* gene was used as a BLASTN query against a subset of T142 and T149 Newbler contigs previously allocated to the *Bathycoccus* genome using the BLASTX approach (see above). This subset was used in order to focus only in similarities against *Bathycoccus* genome. Contigs with a positive match were selected based on e-value (< e^−100^) and visual inspection of the alignment. Contigs providing best match were aligned against the reference gene and nucleotide similarity was determined.

### Global Similarity between the Three Genomes

We used Geneious to annotate assemblies of T142 and T149 against *Bathycoccus* for areas with high coverage (≥10×). For the three larger chromosomes (1 to 3), the *Bathycoccus* sequences and the T142 and T149 consensus were aligned with the MAFTT plugin under Geneious. We discarded all regions from the alignment for which either T142 or T149 had a genome coverage below 10× based on the annotation made by Geneious. We computed the percent of identical bases across the three genomes and for each pair of genome. We then performed the same computation both on coding (CDS) and non-coding regions.

### Genotypes

Assembly of small regions of a few selected individual single-copy genes was manually analyzed under Geneious to assess the minimum number of haplotypes present in the two samples. Briefly, reads were ordered based on their similarity/differences to the reference RCC1105. Several reads were considered to correspond to one genotype when they presented similar changes at least two positions (see Figure 3 for an example).

### Analysis of the rRNA Operon

First, the second RCC1105 rRNA operon on chromosome_11 (RCC1105 has two identical rRNA operons located on this chromosome, the first one in the reverse direction and the second one in the forward direction) was used to search by BLASTN Geneious contigs containing the operon. The two contigs T142_109 and T149_486 were aligned with the RCC1105 rRNA operon using Geneious aligner (Figure S2). Then, in order to analyze genotype variability, we directly assembled T142 and T149 reads to the RCC1105 rRNA operon using the Geneious assembler (medium sensitivity): 290 and 167 reads were assembled for T142 and T149, respectively (Figure S3). Single nucleotide variability was determined using Geneious based on a minimum coverage of 5 reads, the presence of at least two reads bearing the same variation and a minimum frequency of 15% (Table S7).

Sequences of Geneious contigs are available from EMBL-EBI database under accession numbers CAFX01000001-CAFX01015049 (T142) and CAFY01000001-CAFY01022174 (T149). Sequence data for the genome of *Bathycoccus prasinos* RCC 1105 have been deposited to EMBL/GenBank under accession numbers FO082258 (Mitochondrion), FO082259 (Chloroplast), FO082278 (Chromosome1), FO082277 (Chromosome 2), FO082276 (Chromosome 3), FO082275 (Chromosome 4), FO082274 (chromosome 5), FO082273 (Chromosome 6), FO082272 (Chromosome 7), FO082271 (Chromosome 8), FO082270 (Chromosome 9), FO082269 (Chromosome 10), FO082268 (Chromosome 11), FO082267 (Chromosome 12), FO082266 (Chromosome 13), FO082265 (Chromosome 14), FO082264 (Chromosome 15), FO082263 (Chromosome 16), FO082262 (Chromosome 17), FO082261 (Chromosome 18), FO082260 (Chromosome 19). The annotation of the genome can be found on the BOGAS web site (http://bioinformatics.psb.ugent.be/webtools/bogas/).

## Supporting Information

Figure S1 **Relationship between numbers of reads assigned to each** ***B. prasinos*** **RCC1105 chromosome based on BLASTX-based analysis of contigs (see Materials and Methods for details) or direct assembly with Geneious.**
(TIF)

Figure S2 **Structure of the rRNA operon.** (A) Alignment of the *B. prasinos* RCC1105 rRNA operon (forward copy) with the two contigs T142_109 and T149_486 that contain it. Both contigs do not contain the 433 bp SSU rRNA gene intron that characterizes RCC1105. Contig T149_486 appears to contain a 468 bp intron at the end of the LSU rRNA gene. (B) Detail of the putative intron at the end of the LSU rRNA gene of T149_486.
(TIF)

Figure S3 **Genotypic variability of the rRNA operon.** T142 and T149 reads were directly assembled with Geneious to the *B. prasinos* RCC1105 rRNA operon. (A) Individual reads for sample T142 in the region of the SSU rRNA intron present in the genome of RCC 1105. The arrow points to a read with a sequence nearly identical to the intron sequence. (B) Individual reads for sample T142 at the end of the LSU rRNA gene. Ten out of 15 reads have a different sequence suggesting the presence of an intron. (C) Idem for sample T149 at the end of the LSU rRNA gene. Only one read has a sequence suggesting the presence of an intron which sequence is similar that found in sample T142.
(TIF)

Figure S4 **Percent of false assignment of** ***O. ‘lucimarinus*** **’ proteins based on Best BLAST hit (BBH, blue line) or after filtration with gene-specific thresholds for identity and alignment length (FA, red line) as a function of read length (see Materials and Methods for details).**
(TIF)

Table S1 **SSU rRNA genes detected in Geneious contigs for samples T142 and T149.**
(PDF)

Table S2 **Assignment of reads and Geneious contigs for samples T142 and T149 to phylogenetic groups based on BLASTN search of a subset of the nr GenBank database, analyzed by MEGAN (see Materials and Methods for details).** The contribution of the best represented groups and of the eukaryotic groups expected to be present in the samples are detailed.
(PDF)

Table S3 **Assignment of Geneious contigs for samples T142 and T149 to reference genomes of microalgae, bacteria and viruses based on a BLASTX-based algorithm (see Materials and Methods for details).**
(PDF)

Table S4 **Assignment of Geneious contigs for samples T142 and T149 to specific chromosomes of** ***B. prasinos*** **RCC1105 based on a BLASTX-based algorithm (see Materials and Methods for details).** An estimate of coverage was computed as the ratio between the total length of contigs assigned to one chromosome and the length of this chromosome. This value may exceed 100% since some contigs may overlap each other.
(PDF)

Table S5 **Similarity between specific genes from selected gene classes of** ***B. prasinos*** **RCC1105 genome and T142 and T149 Newbler contigs (see Materials and Methods for details).**
(PDF)

Table S6 **Estimation of the number of major haplotypes and of their frequency for selected regions of genes with a high read coverage in both samples.**
(PDF)

Table S7 **Genotype variability within the 5,646 bp rRNA operon. Positions are given along chromosome 11.** Localisation of helix 3 of ITS2 follows Marin and Melkonian [39].
(PDF)

Table S8 **Assignment of reads for Pacific picoeukaryote samples T142 to individual chromosomes of** ***O.’ lucimarinus’*** **using Geneious Assembler (see Materials and Methods for details).**
(PDF)

Table S9 **List of reference genomes used for BLASTX analysis of contigs.**
(PDF)
