# Supplementary figures and images for: Metagenomes of the Picoalga Bathycoccus from the Chile Coastal Upwelling
Source: PLoS One. 2012 Jun 22;7(6):e39648. doi: 10.1371/journal.pone.0039648 (PMC3382182; doi:10.1371/journal.pone.0039648)

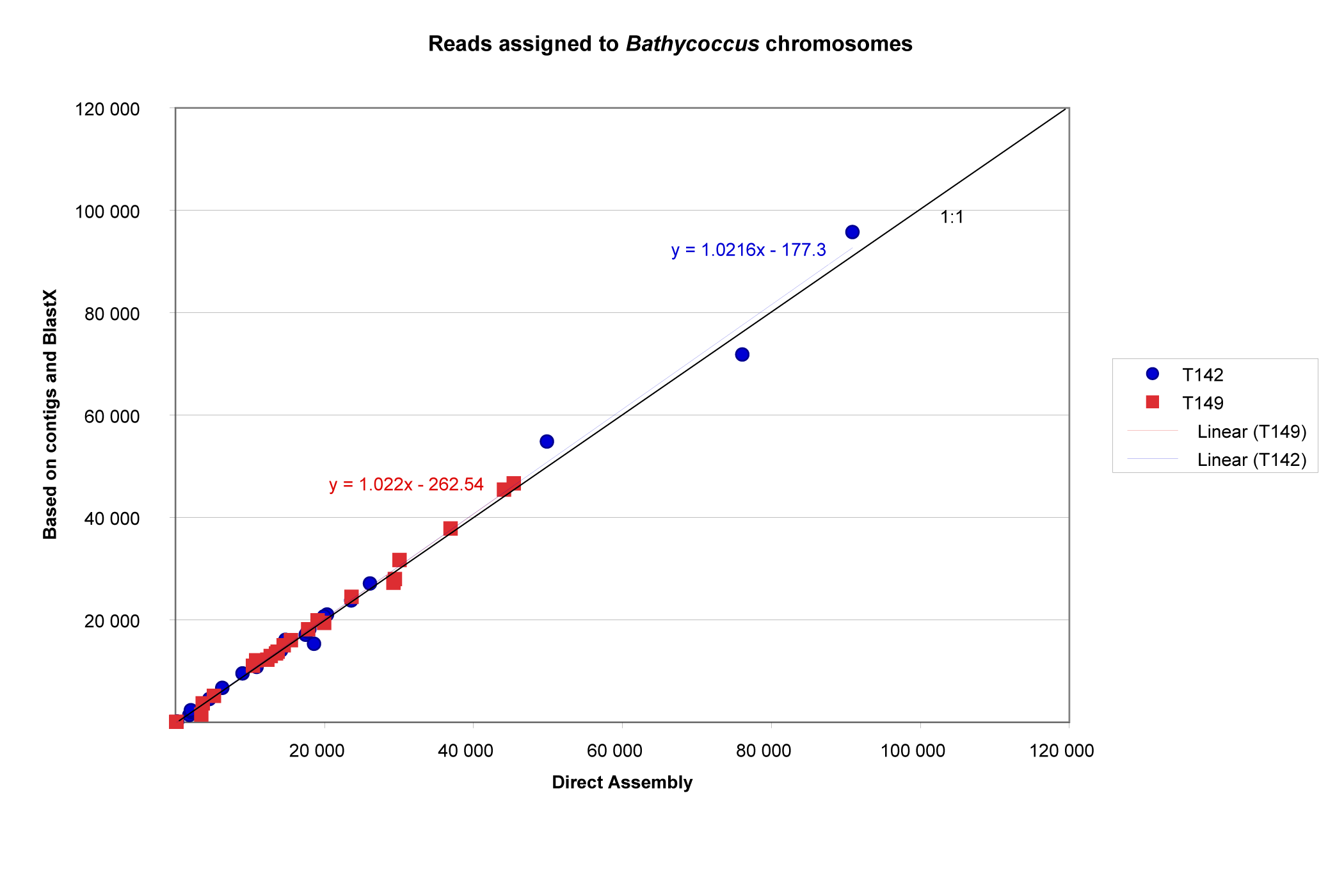

Supplement: Figure S1 — Relationship between numbers of reads assigned to each B. prasinos RCC1105 chromosome based on BLASTX-based analysis of contigs (see Materials and Methods for details) or direct assembly with Geneious. (TIF) [file pone.0039648.s001.tif]

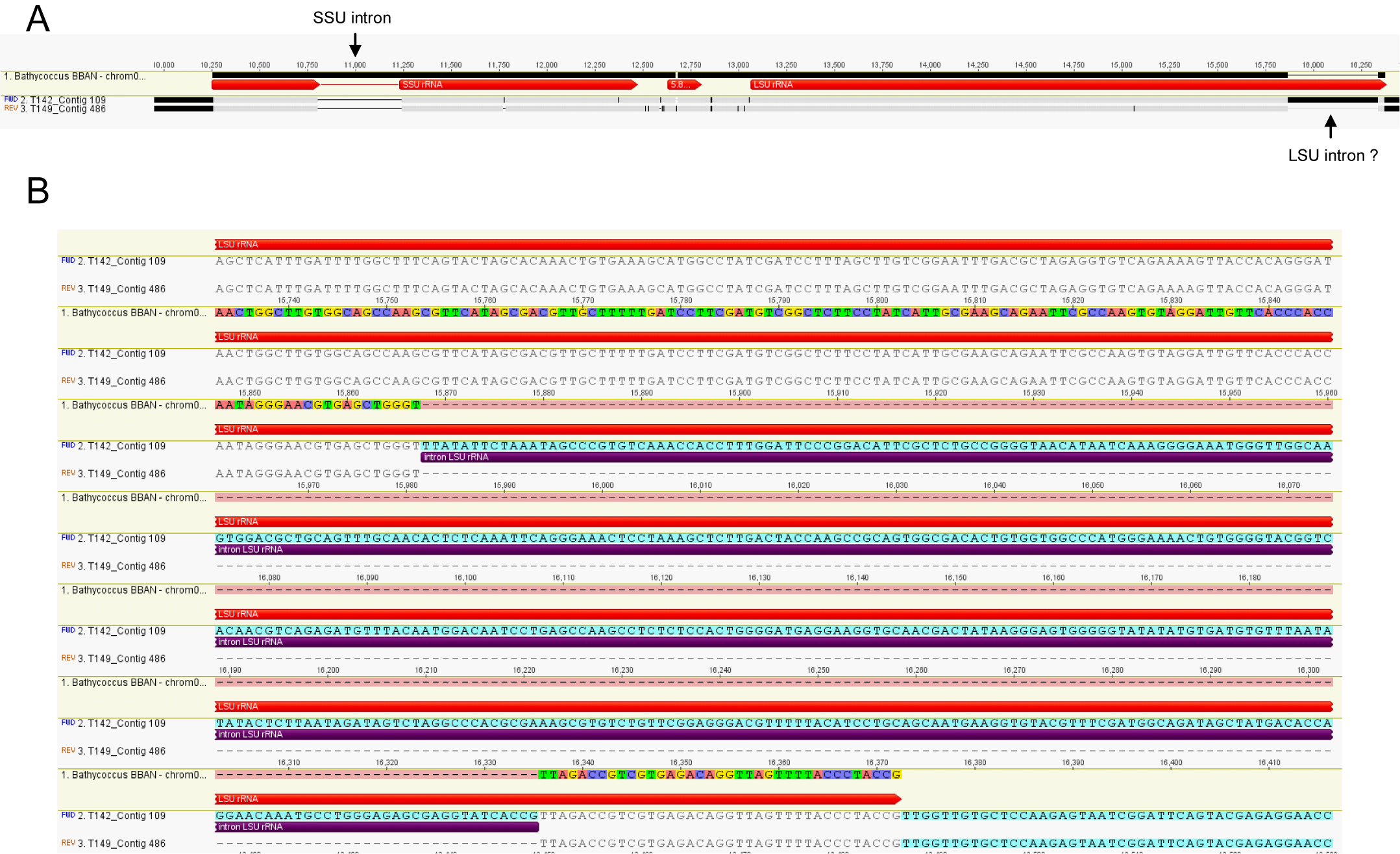

Supplement: Figure S2 — Structure of the rRNA operon. (A) Alignment of the B. prasinos RCC1105 rRNA operon (forward copy) with the two contigs T142_109 and T149_486 that contain it. Both contigs do not contain the 433 bp SSU rRNA gene intron that characterizes RCC1105. Contig T149_486 appears to contain a 468 bp intron at the end of the LSU rRNA gene. (B) Detail of the putative intron at the end of the LSU rRNA gene of T149_486. (TIF) [file pone.0039648.s002.tif]

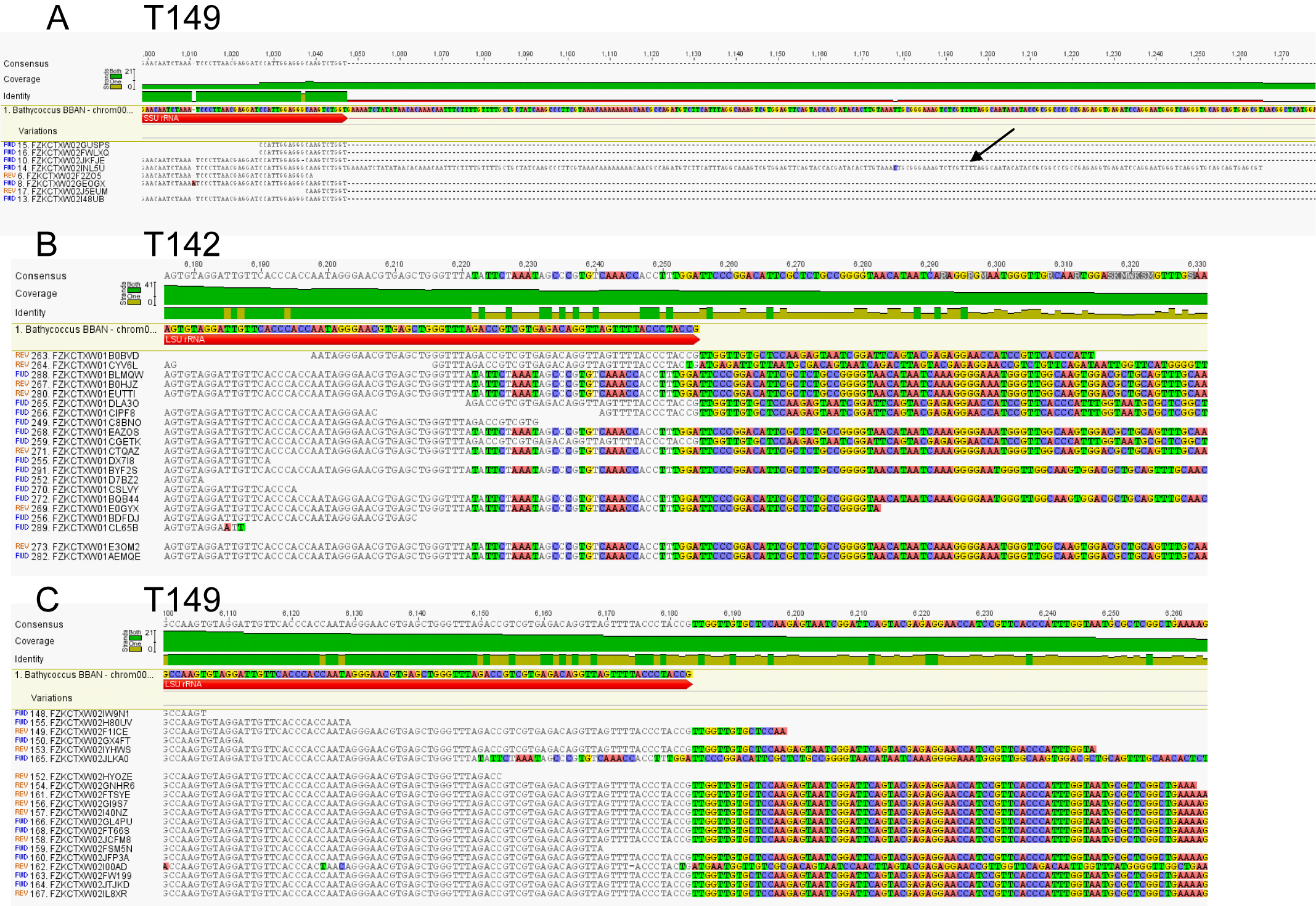

Supplement: Figure S3 — Genotypic variability of the rRNA operon. T142 and T149 reads were directly assembled with Geneious to the B. prasinos RCC1105 rRNA operon. (A) Individual reads for sample T142 in the region of the SSU rRNA intron present in the genome of RCC 1105. The arrow points to a read with a sequence nearly identical to the intron sequence. (B) Individual reads for sample T142 at the end of the LSU rRNA gene. Ten out of 15 reads have a different sequence suggesting the presence of an intron. (C) Idem for sample T149 at the end of the LSU rRNA gene. Only one read has a sequence suggesting the presence of an intron which sequence is similar that found in sample T142. (TIF) [file pone.0039648.s003.tif]

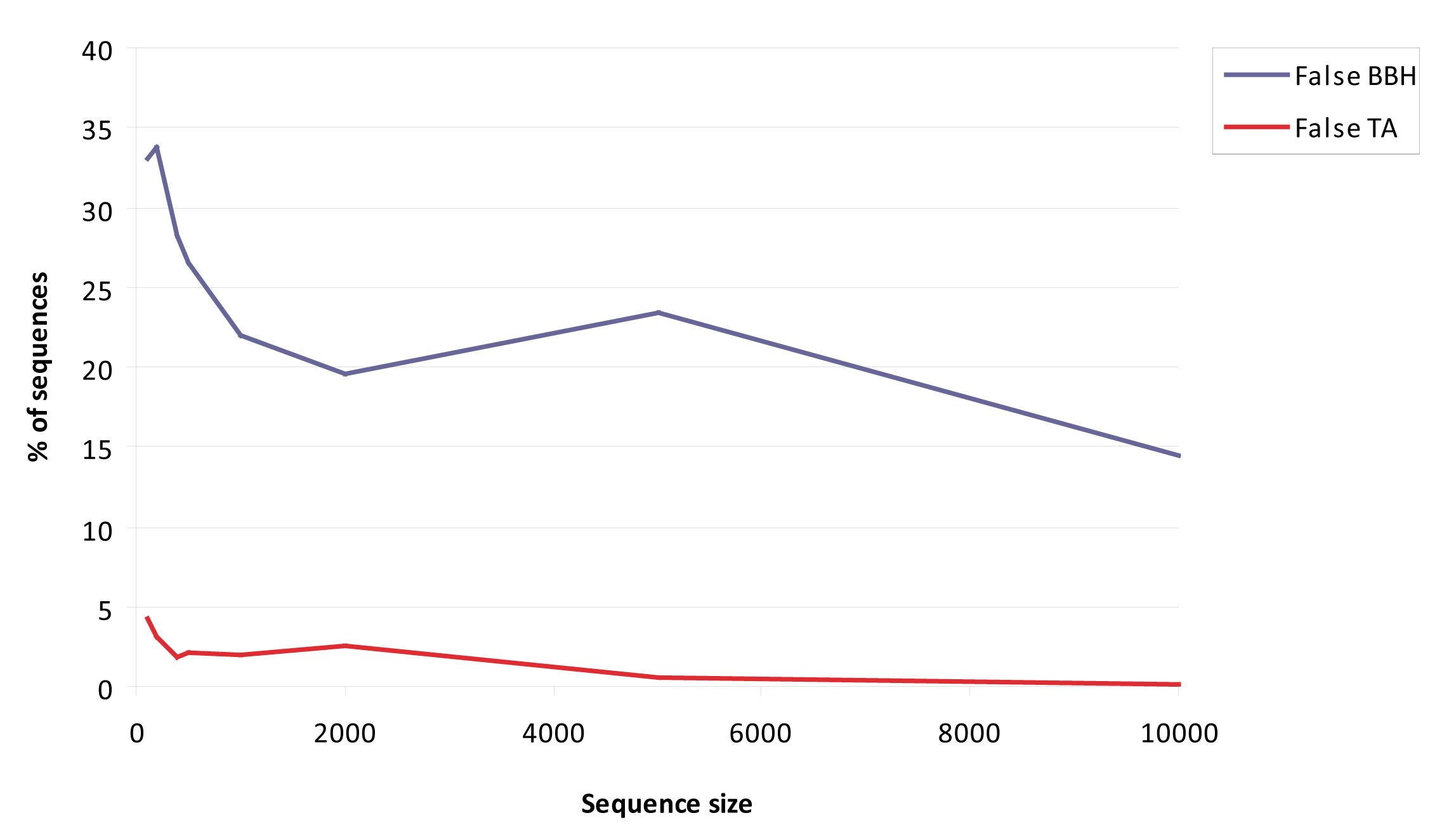

Supplement: Figure S4 — Percent of false assignment of O. ‘lucimarinus ’ proteins based on Best BLAST hit (BBH, blue line) or after filtration with gene-specific thresholds for identity and alignment length (FA, red line) as a function of read length (see Materials and Methods for details). (TIF) [file pone.0039648.s004.tif]
