# Supplementary material for: Metagenomes of the Picoalga Bathycoccus from the Chile Coastal Upwelling
Source: PLoS One. 2012 Jun 22;7(6):e39648. doi: 10.1371/journal.pone.0039648 (PMC3382182; doi:10.1371/journal.pone.0039648)
Supplement: Table S1 — SSU rRNA genes detected in Geneious contigs for samples T142 and T149. (PDF) [file pone.0039648.s005.pdf]

Table S1

Kingdom	Division	Class	Localisation	T142	Clade or genus		T149	Clade or genus
Bacteria	Bacteroidetes	Flavobacteria					1	*Zunongwangia*
Bacteria	Deferribacteres	Deferribacterales					1	SAR406
Bacteria	Proteobacteria	Alphaproteobacteria		4	*Roseobacter, Pelagibacter* (SAR11)		5	*Roseobacter, Pelagibacter* (SAR11)
Bacteria	Proteobacteria	Gammaproteobacteria		2	*Marinomonas, Pseudospirillum*		3	SAR86, SAR92
Bacteria	Proteobacteria	Deltaproteobacteria		1	*Bacteriovorax*		3	*Bacteriovorax*
Eukaryota	Chlorophyta	Mamelliophyceae	nuclear	1	*Bathycoccus*		1	*Bathycoccus*
Eukaryota	Stremenopiiles	Dictyochophyceae	nuclear				1	*Apedinella*
Eukaryota	Chlorophyta	Mamelliophyceae	plastid	2	*Bathycoccus, Micromonas*		1	*Micromonas*
Eukaryota	Chlorophyta	Mamelliophyceae	mitochondrion	4	*Ostreococcus, Micromonas*		2	*Micromonas*
Eukaryota	Stremenopiiles	Chrysophyceae	mitochondrion				2	*Chrysodidymus*
			**Total**	**14**			**20**	
Only hits > 200 bp wre considered								
