# Supplementary material for: Metagenomes of the Picoalga Bathycoccus from the Chile Coastal Upwelling
Source: PLoS One. 2012 Jun 22;7(6):e39648. doi: 10.1371/journal.pone.0039648 (PMC3382182; doi:10.1371/journal.pone.0039648)
Supplement: Table S2 — Assignment of reads and Geneious contigs for samples T142 and T149 to phylogenetic groups based on BLASTN search of a subset of the nr GenBank database, analyzed by MEGAN (see Materials and Methods for details). The contribution of the best represented groups and of the eukaryotic groups expected to be present in the samples are detailed. (PDF) [file pone.0039648.s006.pdf]

Table S2

	T142 reads	T142 % of total		T149 reads	T149 % of total		T142 Contigs Geneious	T142 % of total		T149 Contigs Geneious	T149 % of total
Global affiliation											
Archaea	1 301	0.19%		1 773	0.26%		80	0.35%		105	0.30%
Bacteria	54 580	8.13%		52 192	7.77%		4 175	18.01%		6 481	18.60%
- *Proteobacteria*	32 098	*4.78%*		31 564	*4.70%*		*2 646*	*11.41%*		*4 289*	*12.31%*
--- *Candidatus Pelagibacter*	11 764	*1.75%*		9 941	*1.48%*		*1 331*	*5.74%*		*1 911*	*5.49%*
--- *Bacteriovorax marinus*	1 427	*0.21%*		2 473	*0.37%*		*234*	*1.01%*		*427*	*1.23%*
Eukaryota	216 862	32.31%		202 366	30.12%		7 550	32.56%		10 653	30.58%
- *Viridiplantae*	96 245	*14.34%*		100 052	*14.89%*		*3 322*	*14.33%*		*5 123*	*14.70%*
-- *Mamiellales*	39 047	*5.82%*		40 700	*6.06%*		*1 470*	*6.34%*		*2 260*	*6.49%*
--- *Ostreococcus*	28 022	*4.17%*		28 460	*4.24%*		*1 072*	*4.62%*		*1 567*	*4.50%*
--- *Micromonas*	8 367	*1.25%*		9 787	*1.46%*		*303*	*1.31%*		*530*	*1.52%*
--- *Bathycoccus*	125	*0.02%*		184	*0.03%*		*10*	*0.04%*		*28*	*0.08%*
- *Stramenopiles*	2 907	*0.43%*		4 098	*0.61%*		*88*	*0.38%*		*137*	*0.39%*
- *Haptophyta*	983	*0.15%*		719	*0.11%*		*21*	*0.09%*		*29*	*0.08%*
- *Alveolata*	2 102	*0.31%*		2 414	*0.36%*		*87*	*0.38%*		*114*	*0.33%*
Viruses	1 186	0.18%		1 065	0.16%		70	0.30%		70	0.20%
Unknown	397 320	59.19%		414 436	61.69%		11 312	48.79%		17 530	50.32%
Total number of reads or contigs	671 249			671 832			23 187			34 839	
Total number of taxa (species level)	1 646			1 907			260			413	
