# Supplementary material for: Metagenomes of the Picoalga Bathycoccus from the Chile Coastal Upwelling
Source: PLoS One. 2012 Jun 22;7(6):e39648. doi: 10.1371/journal.pone.0039648 (PMC3382182; doi:10.1371/journal.pone.0039648)
Supplement: Table S3 — Assignment of Geneious contigs for samples T142 and T149 to reference genomes of microalgae, bacteria and viruses based on a BLASTX-based algorithm (see Materials and Methods for details). (PDF) [file pone.0039648.s007.pdf]

Table S3

		T142						T149						Mean [2]	
		contigs		nucleotides		reads		contigs		nucleotides		reads		contigs	reads
**Affiliation**		**N**	**%**	**N**	**%**	**N**	**%**	**N**	**%**	**N**	**%**	**N**	**%**	**%**	**%**
Eukaryota															
- Viridiplantae	*Bathycoccus* [1]	7 428	32.04%	10 702 436	46.72%	450 621	71.10%	10 788	30.97%	18 370 708	52.57%	415 842	68.48%	31.50%	69.79%
	Micromonas	78	0.34%	48 196	0.21%	280	0.04%	199	0.57%	131 733	0.38%	903	0.15%	0.45%	0.10%
	Ostreococcus	110	0.47%	77 936	0.34%	416	0.07%	87	0.25%	79 871	0.23%	1 106	0.18%	0.36%	0.12%
	Mamiellophyceae	1 054	4.55%	1 108 676	4.84%	31 868	5.03%	1 716	4.93%	1 757 860	5.03%	30 395	5.01%	4.74%	5.02%
	chloroplast Mamiellophyceae	1	0.00%	253	0.00%	2	0.00%	10	0.03%	5 910	0.02%	27	0.00%	0.02%	0.00%
	mitochondria Mamiellophyceae	27	0.12%	27 146	0.12%	283	0.04%	26	0.07%	22 021	0.06%	543	0.09%	0.10%	0.07%
- Stramenopiles	*Aureococcus*	4	0.02%	2 172	0.01%	12	0.00%	4	0.01%	3 001	0.01%	16	0.00%	0.01%	0.00%
	Fragilariopsis	2	0.01%	651	0.00%	5	0.00%							0.00%	0.00%
	Phaeodactylum	1	0.00%	403	0.00%	2	0.00%	1	0.00%	449	0.00%	4	0.00%	0.00%	0.00%
	Thalassiosira	3	0.01%	2 762	0.01%	33	0.01%	3	0.01%	2 338	0.01%	8	0.00%	0.01%	0.00%
- Haptophyta	*Emiliania*							1	0.00%	803	0.00%	2	0.00%	0.00%	0.00%
- Fungi	*Candida*	1	0.00%	479	0.00%	3	0.00%							0.00%	0.00%
	Saccharomyces	1	0.00%	279	0.00%	3	0.00%							0.00%	0.00%
Bacteria	*Bacillus*	1	0.00%	940	0.00%	5	0.00%	1	0.00%	779	0.00%	7	0.00%	0.00%	0.00%
	Marinobacter	15	0.06%	9 237	0.04%	47	0.01%	28	0.08%	14 836	0.04%	104	0.02%	0.07%	0.01%
	Candidatus Pelagibacter	805	3.47%	634 493	2.77%	7 426	1.17%	1 132	3.25%	724 186	2.07%	5 732	0.94%	3.36%	1.06%
	Planctomyces	2	0.01%	1 298	0.01%	7	0.00%	3	0.01%	1 380	0.00%	7	0.00%	0.01%	0.00%
	Roseobacter	47	0.20%	41 419	0.18%	353	0.06%	127	0.36%	72 807	0.21%	407	0.07%	0.28%	0.06%
Viruses	Prasinovirus	5	0.02%	4 084	0.02%	23	0.00%	17	0.05%	9 510	0.03%	57	0.01%	0.04%	0.01%
Unknown	Unaffiliated	12 653	54.57%	9 755 641	42.59%	136 779	21.58%	19 329	55.48%	13 189 413	37.74%	147 715	24.33%	55.03%	22.95%
	No hit	949	4.09%	489 372	2.14%	5 612	0.89%	1 367	3.92%	560 056	1.60%	4 361	0.72%	4.01%	0.80%
	Total	23 187		22 907 873		633 780		34 839		34 947 661		607 236			

[1] Includes also plastid and mitochondrial genomes

[2] Average of values for T142 and T149
