# Supplementary material for: Metagenomes of the Picoalga Bathycoccus from the Chile Coastal Upwelling
Source: PLoS One. 2012 Jun 22;7(6):e39648. doi: 10.1371/journal.pone.0039648 (PMC3382182; doi:10.1371/journal.pone.0039648)
Supplement: Table S4 — Assignment of Geneious contigs for samples T142 and T149 to specific chromosomes of B. prasinos RCC1105 based on a BLASTX-based algorithm (see Materials and Methods for details). An estimate of coverage was computed as the ratio between the total length of contigs assigned to one chromosome and the length of this chromosome. This value may exceed 100% since some contigs may overlap each other. (PDF) [file pone.0039648.s008.pdf]

Table S4

				T142	T142	T142	T142		T149	T149
		**Length**	**GC%**	**Contigs**	**Nucleotides**	**Reads**	**Approximate Coverage**		**Contigs**	**Nucleotic**
Chromosome_final	Chromosome_draft	**bp**	**%**	**#**	**bp**	**#**			**#**	**bp**
chromosome_1	Bathy_chrom000	1 352 574	48.87%	557	845 991	17 094	63%		1 017	1 733 5
chromosome_2	Bathy_chrom001	1 122 692	48.55%	792	1 070 483	71 791	95%		839	1 557 7
chromosome_3	Bathy_chrom002	1 089 374	48.62%	536	837 337	23 794	77%		824	1 488 4
chromosome_4	Bathy_chrom003	1 037 991	48.29%	441	604 045	14 038	58%		723	1 161 0
chromosome_5a	Bathy_chrom012	550 167	48.34%	197	249 530	4 533	45%		372	662 1
chromosome_5b	Bathy_chrom016	467 783	48.58%	164	185 418	1 975	40%		371	610 3
chromosome_6	Bathy_chrom004	989 707	48.30%	505	769 365	17 703	78%		772	1 278 4
chromosome_7	Bathy_chrom005	955 054	48.42%	621	810 304	54 820	85%		729	1 204 8
chromosome_8	Bathy_chrom006	937 610	48.54%	390	523 141	10 784	56%		729	1 231 2
chromosome_9	Bathy_chrom007	895 347	48.51%	410	712 327	18 122	80%		650	1 117 4
chromosome_10	Bathy_chrom008	794 148	48.38%	413	634 920	20 988	80%		627	1 061 4
chromosome_11	Bathy_chrom009	741 502	48.62%	394	651 024	27 057	88%		478	798 3
chromosome_12a	Bathy_chrom019	201 229	47.52%	75	123 219	2 304	61%		149	228 4
chromosome_12b	Bathy_chrom014	511 334	48.55%	300	493 261	20 640	96%		438	747 3
chromosome_13	Bathy_chrom010	706 576	48.54%	609	687 835	95 748	97%		426	796 1
chromosome_14	Bathy_chrom011-50	662 304	42.24%	176	185 049	1 412	28%		344	477 9
chromosome_15	Bathy_chrom013	519 535	48.13%	205	340 695	6 691	66%		374	642 6
chromosome_16	Bathy_chrom015	481 036	48.08%	207	299 394	16 053	62%		358	644 6
chromosome_17	Bathy_chrom017-28	465 570	47.66%	232	370 091	15 314	79%		293	524 2
chromosome_18	Bathy_chrom018	310 170	46.97%	171	281 730	9 550	91%		218	332 1
chromosome_19	Bathy_chrom020	146 238	41.65%	9	4 926	22	3%		4	4 9
Chloroplast	Bathy_chrom021 – plastid	54 761	41.12%	2	621	4	1%		5	2 9
Mitochondrion	Bathy_chrom024 – mito	42 168	39.97%	18	17 326	157	41%		17	27 7
contigs_others	Bathy_chrom others	1 201 689		4	4 404	27	0%		31	36 3
	Total for Bathycoccus	16 236 559		7 428	10 702 436	450 621			10 788	18 370 7
	Total sample			23 187	22 907 873	633 780			34 839	34 947 6
