# Supplementary material for: Metagenomes of the Picoalga Bathycoccus from the Chile Coastal Upwelling
Source: PLoS One. 2012 Jun 22;7(6):e39648. doi: 10.1371/journal.pone.0039648 (PMC3382182; doi:10.1371/journal.pone.0039648)
Supplement: Table S5 — Similarity between specific genes from selected gene classes of B. prasinos RCC1105 genome and T142 and T149 Newbler contigs (see Materials and Methods for details). (PDF) [file pone.0039648.s009.pdf]

Table S5

Category	Pathway	Gene	Gene Product	Gene in bathycoccus	T-142_contig	T-142_bases	T-142_bases similar	T-142_identity	T-149_contig	T-149_bases	T-149_bases similar	T-149_identity
cell cycle	cell cycle	APC1	Anaphase-promoting complex, subunit 1	Bathy05g02870	contig05822	878	887	99.0%	contig06470	3868	3917	98.7%
cell cycle	cell cycle	APC1	Anaphase-promoting complex, subunit 1	Bathy05g02871	contig08672	478	490	97.6%				
cell cycle	cell cycle	APC1	Anaphase-promoting complex, subunit 1	Bathy05g02872	contig08460	369	375	98.4%				
cell cycle	cell cycle	APC1	Anaphase-promoting complex, subunit 1	Bathy05g02873	contig14047	355	363	97.8%				
cell cycle	cell cycle	APC10	Anaphase-promoting complex, subunit 10	Bathy11g03140	contig02550	578	587	98.5%	contig26149	769	779	98.7%
cell cycle	cell cycle	APC10	Anaphase-promoting complex, subunit 10	Bathy11g03141	contig16518	570	581	98.1%	contig26345	767	780	98.3%
cell cycle	cell cycle	APC10	Anaphase-promoting complex, subunit 10	Bathy11g03142	contig02549	304	306	99.3%	contig27101	212	212	100.0%
cell cycle	cell cycle	APC10	Anaphase-promoting complex, subunit 10	Bathy11g03142	contig17207	245	260	94.2%				
cell cycle	cell cycle	APC11	Anaphase promoting complex, subunit 11	Bathy13g00100	contig00353	411	417	98.6%	contig25375	309	312	99.0%
cell cycle	cell cycle	APC2	Anaphase promoting complex, subunit 2	Bathy13g02800	contig07303	538	541	99.4%	contig00545	3535	3577	98.8%
cell cycle	cell cycle	APC2	Anaphase promoting complex, subunit 2	Bathy13g02800	contig08634	749	759	98.7%				
cell cycle	cell cycle	APC3/CDC27	Anaphase promoting complex, subunit 3	Bathy05g00120	contig04775	2711	2751	98.5%	contig02027	2859	2907	98.3%
cell cycle	cell cycle	APC3/CDC27	Anaphase promoting complex, subunit 3	Bathy05g00121	contig04774	108	111	97.3%				
cell cycle	cell cycle	APC4	Anaphase promoting complex, subunit 4	Bathy03g02330	contig05714	1253	1262	99.3%	contig23302	1866	1879	99.3%
cell cycle	cell cycle	APC4	Anaphase promoting complex, subunit 4	Bathy03g02331	contig09579	531	538	98.7%	contig12493	252	257	98.1%
cell cycle	cell cycle	APC4	Anaphase promoting complex, subunit 4	Bathy03g02331					contig12490	168	171	98.2%
cell cycle	cell cycle	APC5	Anaphase promoting complex, subunit 5	Bathy05g04700	contig07386	134	138	97.1%	contig03199	1809	1828	99.0%
cell cycle	cell cycle	APC5	Anaphase promoting complex, subunit 5	Bathy05g04701					contig00617	1645	1695	97.1%
cell cycle	cell cycle	APC6/CDC16	Anaphase promoting complex, subunit 6	Bathy01g00910	contig06073	986	989	99.7%	contig03315	2318	2351	98.6%
cell cycle	cell cycle	APC7	Anaphase promoting complex, subunit 7	Bathy04g00320					contig01032	1998	2007	99.6%
cell cycle	cell cycle	APC8/CDC23	Anaphase promoting complex, subunit 8	Bathy02g03140	contig01965	409	413	99.0%	contig25818	401	405	99.0%
cell cycle	cell cycle	APC8/CDC23	Anaphase promoting complex, subunit 8	Bathy02g03141	contig17313	391	413	94.7%	contig04033	385	407	94.6%
cell cycle	cell cycle	APC8/CDC23	Anaphase promoting complex, subunit 8	Bathy02g03141	contig16426	1545	1552	99.5%	contig03910	1556	1560	99.7%
cell cycle	cell cycle	CDC20/CDH1/AMA1	Cell division cycle protein 20	Bathy08g04250	contig16405	1405	1419	99.0%	contig25096	1405	1419	99.0%
cell cycle	cell cycle	CDH1-Ccs52	Cell cycle switch protein	Bathy04g02240					contig00083	1857	1915	97.0%
cell cycle	cell cycle	CDKA	Cell cycle dependent kinase A	Bathy05g04860					contig03194	915	923	99.1%
cell cycle	cell cycle	CDKB	Cell cycle dependent kinase B	Bathy02g02440					contig02290	883	888	99.4%
cell cycle	cell cycle	CDKC	Cell cycle dependent kinase C	Bathy01g03640	contig00191	1879	1914	98.2%	contig00739	1879	1915	98.1%
cell cycle	cell cycle	CDKD	Cell cycle dependent kinase D	Bathy02g04380	contig00139	1219	1243	98.1%	contig01473	1217	1240	98.1%
cell cycle	cell cycle	CKS	Cyclin-dependent kinases regulatory subunit	Bathy02g03570					contig26714	139	140	99.3%
cell cycle	cell cycle	CKS	Cyclin-dependent kinases regulatory subunit	Bathy02g03571					contig00836	138	140	98.6%
cell cycle	cell cycle	CycB	Cyclin B	Bathy15g02230					contig25359	1092	1100	99.3%
cell cycle	cell cycle	CycH	Cyclin H	Bathy09g02290	contig02310	782	798	98.0%	contig23658	964	980	98.4%
cell cycle	cell cycle	CycH	Cyclin H	Bathy09g02291	contig02311	120	121	99.2%				
cell cycle	cell cycle	CycH	Cyclin H	Bathy09g02292	contig02315	106	108	98.1%				
cell cycle	cell cycle	CycT	Cyclin T	Bathy14g02190					contig09355	144	144	100.0%
cell cycle	cell cycle	CycT	Cyclin T	Bathy14g02190					contig09357	136	138	98.6%
cell cycle	cell cycle	DEL	DP-E2F	Bathy02g04850					contig01001	2482	2528	98.2%
cell cycle	cell cycle	DEL	DP-E2F	Bathy02g04850					contig05170	172	175	98.3%
cell cycle	cell cycle	DP	E2F dimerization partner	Bathy11g01110	contig05135	1220	1245	98.0%	contig00270	2201	2227	98.8%
cell cycle	cell cycle	DP	E2F dimerization partner	Bathy11g01111	contig05138	885	902	98.1%				
cell cycle	cell cycle	E2F	Transcription factor E2F	Bathy14g02250					contig09864	373	374	99.7%
cell cycle	cell cycle	E2F	Transcription factor E2F	Bathy14g02251					contig09865	308	310	99.4%
cell cycle	cell cycle	RB	Retinoblastoma	Bathy16g01700					contig05275	1062	1078	98.5%
cell cycle	cell cycle	RB	Retinoblastoma	Bathy16g01701					contig07813	928	936	99.1%
**cell cycle**	**Mean**							**98.2%**				**98.7%**
Photosynthesis	ATP synthase	ATPC	H+-or Na+-translocating ATPase superfamily	Bathy02g04790					contig01022	975	981	99.4%
Photosynthesis	ATP synthase	ATPD	H+-or Na+-translocating ATPase superfamily	Bathy02g04460	contig16796	543	547	99.3%	contig00551	543	547	99.3%
Photosynthesis	ATP synthase	ATPG	H+-or Na+-translocating ATPase superfamily	Bathy01g00780	contig01842	518	526	98.5%	contig01128	180	182	98.9%
Photosynthesis	Calvin Cycle	FBAI.1	Fructose bisphosphate aldolase class i	Bathy06g02350					contig05729	1022	1036	98.6%
Photosynthesis	Calvin Cycle	FbPase (3)	Fructose-1,6-bisphosphatase	Bathy12g03440					contig10792	1108	1122	98.8%
Photosynthesis	Calvin Cycle	FbPase (2)	Fructose-1,6-bisphosphatase	Bathy10g04130	contig00747	1061	1065	99.6%				
Photosynthesis	Calvin Cycle	FbPase (2a)	Fructose-1,6-bisphosphatase	Bathy06g03760	contig17214	1620	1642	98.7%	contig25656	1180	1192	99.0%
Photosynthesis	Calvin Cycle	FbPase (2a)	Fructose-1,6-bisphosphatase	Bathy06g03760	contig16753	629	634	99.2%	contig26255	1164	1190	97.8%
Photosynthesis	Calvin Cycle	FbPase (2a)	Fructose-1,6-bisphosphatase	Bathy06g03760	contig00101	618	632	97.8%	contig03870	1001	1015	98.6%
Photosynthesis	Calvin Cycle	FbPase (2a)	Fructose-1,6-bisphosphatase	Bathy06g03760	contig16339	390	392	99.5%	contig02618	458	461	99.3%
Photosynthesis	Calvin Cycle	FbPase (2a)	Fructose-1,6-bisphosphatase	Bathy06g03760	contig17279	269	284	94.7%	contig24485	337	353	95.5%

Table S5

Category	Pathway	Gene	Gene Product	Gene in bathycoccus	T-142_contig	T-142_bases	T-142_bases similar	T-142_identity	T-149_contig	T-149_bases	T-149_bases similar	T-149_identity
Photosynthesis	Calvin Cycle	FbPase (1)	Fructose-1,6-bisphosphatase	Bathy14g00390	contig04560	558	561	99.5%	contig26365	1160	1161	99.9%
Photosynthesis	Calvin Cycle	FbPase (1)	Fructose-1,6-bisphosphatase	Bathy14g00390	contig03279	183	183	100.0%				
Photosynthesis	Calvin Cycle	G6PDH	Glucose-6-phosphate dehydrogenase	Bathy02g03340	contig03914	1434	1440	99.6%	contig01693	1250	1255	99.6%
Photosynthesis	Calvin Cycle	G6PDH	Glucose-6-phosphate dehydrogenase	Bathy02g03341					contig01700	437	437	100.0%
Photosynthesis	Calvin Cycle	GAPDH	Glyceraldehyde 3-phosphate dehydrogenase	Bathy12g01480	contig00965	1235	1240	99.6%	contig03474	1224	1243	98.5%
Photosynthesis	Calvin Cycle	GAPDH (B subunit)	Glyceraldehyde 3-phosphate dehydrogenase	Bathy01g01370					contig09753	1125	1137	98.9%
Photosynthesis	Calvin Cycle	GAPDH	Glyceraldehyde 3-phosphate dehydrogenase B	Bathy03g01790	contig00381	1079	1083	99.6%	contig03255	708	713	99.3%
Photosynthesis	Calvin Cycle	GAPDH	Glyceraldehyde 3-phosphate dehydrogenase B	Bathy03g01790					contig27354	370	370	100.0%
Photosynthesis	Calvin Cycle	GAPDH	Glyceraldehyde 3-phosphate dehydrogenase B	Bathy03g01790					contig27355	366	369	99.2%
Photosynthesis	Calvin Cycle	GAPDH (A subunit)	Glyceraldehyde 3-phosphate dehydrogenase B	Bathy11g03690	contig04637	564	565	99.8%	contig09755	292	295	99.0%
Photosynthesis	Calvin Cycle	GAPDH (A subunit)	Glyceraldehyde 3-phosphate dehydrogenase B	Bathy11g03690					contig09754	226	226	100.0%
Photosynthesis	Calvin Cycle	PGI	Phosphoglucose isomerase	Bathy13g00670	contig05302	1042	1055	98.8%	contig00246	1668	1685	99.0%
Photosynthesis	Calvin Cycle	PGI	Phosphoglucose isomerase	Bathy13g00670	contig05306	261	261	100.0%	contig06456	124	131	94.7%
Photosynthesis	Calvin Cycle	PGK	Phosphoglycerate kinase	Bathy05g00620					contig24679	217	217	100.0%
Photosynthesis	Calvin Cycle	PGK	Phosphoglycerate kinase	Bathy05g00620					contig26273	931	935	99.6%
Photosynthesis	Calvin Cycle	PGK	Phosphoglycerate kinase	Bathy05g00620					contig24680	164	165	99.4%
Photosynthesis	Calvin Cycle	PGK (2)	Phosphoglycerate kinase	Bathy03g01660	contig01000	1583	1620	97.7%	contig25512	561	570	98.4%
Photosynthesis	Calvin Cycle	PGK (2)	Phosphoglycerate kinase	Bathy03g01660					contig25848	556	569	97.7%
Photosynthesis	Calvin Cycle	PGK (2)	Phosphoglycerate kinase	Bathy03g01660					contig24591	459	465	98.7%
Photosynthesis	Calvin Cycle	PGK (2)	Phosphoglycerate kinase	Bathy03g01660					contig03939	398	401	99.3%
Photosynthesis	Calvin Cycle	PGK (2)	Phosphoglycerate kinase	Bathy03g01660					contig24590	388	395	98.2%
Photosynthesis	Calvin Cycle	PGK (2)	Phosphoglycerate kinase	Bathy03g01660					contig00717	385	396	97.2%
Photosynthesis	Calvin Cycle	PGK (2)	Phosphoglycerate kinase	Bathy03g01660					contig25604	134	135	99.3%
Photosynthesis	Calvin Cycle	PGK (2)	Phosphoglycerate kinase	Bathy03g01660					contig23530	136	139	97.8%
Photosynthesis	Calvin Cycle	PRK	Phosphoribulokinase	Bathy06g04980	contig09828	161	161	100.0%	contig05798	984	990	99.4%
Photosynthesis	Calvin Cycle	PRK	Phosphoribulokinase	Bathy06g04980	contig15338	130	130	100.0%				
Photosynthesis	Calvin Cycle	RPE	Ribulose-phosphate 3-epimerase	Bathy05g01670	contig07500	198	203	97.5%	contig00481	791	798	99.1%
Photosynthesis	Calvin Cycle	RPE	Ribulose-phosphate 3-epimerase	Bathy05g01770					contig00883	572	573	99.8%
Photosynthesis	Calvin Cycle	RPE	Ribulose-phosphate 3-epimerase	Bathy05g01770					contig26670	122	123	99.2%
Photosynthesis	Calvin Cycle	RPE	Ribulose-phosphate 3-epimerase	Bathy05g01770					contig26671	119	123	96.7%
Photosynthesis	Calvin Cycle	rbcL	RuBisCo	Bathy02g03660	contig02294	1748	1772	98.6%	contig06487	657	678	96.9%
Photosynthesis	Calvin Cycle	rbcL	RuBisCo	Bathy02g03660					contig06257	1214	1221	99.4%
Photosynthesis	Calvin Cycle	rbcL	RuBisCo	Bathy02g03660					contig27790	668	678	98.5%
Photosynthesis	Calvin Cycle	SbPase	Sedoheptulose-1,7-bisphosphatase	Bathy07g00360	contig10032	834	838	99.5%	contig02431	1121	1125	99.6%
Photosynthesis	Calvin Cycle	TKL	Transketolase	Bathy17g02080	contig09708	495	497	99.6%	contig04804	1516	1522	99.6%
Photosynthesis	Calvin Cycle	TKL	Transketolase	Bathy17g02081					contig14795	349	351	99.4%
Photosynthesis	Calvin Cycle	TKL	Transketolase	Bathy17g02081					contig04806	113	113	100.0%
Photosynthesis	Calvin Cycle	TPI	Triosephosphate isomerase	Bathy02g05710	contig06558	1246	1259	99.0%	contig00194	962	980	98.2%
Photosynthesis	Calvin Cycle	TPI	Triosephosphate isomerase	Bathy02g05710	contig05351	486	496	98.0%	contig23236	194	194	100.0%
Photosynthesis	Calvin Cycle	TPI	Triosephosphate isomerase	Bathy02g05710	contig06561	276	289	95.5%				
Photosynthesis	Calvin Cycle	TPI	Triosephosphate isomerase	Bathy02g05710	contig06560	127	130	97.7%				
Photosynthesis	Calvin Cycle	TPI	Triosephosphate isomerase	Bathy10g04140	contig00747	792	795	99.6%				
Photosynthesis	Calvin Cycle	TPI	Triosephosphate isomerase	Bathy12g01650	contig02357	1009	1041	96.9%	contig24383	444	456	97.4%
Photosynthesis	Calvin Cycle	TPI	Triosephosphate isomerase	Bathy12g01650					contig26866	434	470	92.3%
Photosynthesis	Calvin Cycle	TPI	Triosephosphate isomerase	Bathy12g01650					contig27185	300	301	99.7%
Photosynthesis	Calvin Cycle	TPI	Triosephosphate isomerase	Bathy12g01650					contig01253	240	241	99.6%
Photosynthesis	Cytochrome b6/f complex	PETC	Cytochrome b6/f complex iron-sulfur subunit	Bathy15g02600	contig08780	123	123	100.0%	contig01794	503	507	99.2%
Photosynthesis	Cytochrome b6/f complex	PETC	Cytochrome b6/f complex iron-sulfur subunit	Bathy15g02600					contig01796	123	123	100.0%
Photosynthesis	Cytochrome b6/f complex	PETD	Cytochrome b6-f complex subunit IV	Bathy02g05300	contig04313	116	116	100.0%	contig27050	523	525	99.6%
Photosynthesis	Cytochrome b6/f complex	PETD	Cytochrome b6-f complex subunit IV	Bathy02g05301					contig24695	116	116	100.0%
Photosynthesis	Cytochrome b6/f complex	PETD	Cytochrome b6-f complex subunit IV	Bathy02g05302					contig27325	115	116	99.1%
Photosynthesis	Cytochrome b6/f complex	PETM	Cytochrome b6/f complex subunit	Bathy08g03640					contig05298	68	68	100.0%
Photosynthesis	Cytochrome b6/f complex	PETN	Cytochrome b6-f complex subunit	Bathy07g04100					contig13868	119	120	99.2%
Photosynthesis	Photosystem I	PSAD	Photosystem I subunit II, cp precursor	Bathy08g00350					contig03855	348	351	99.1%
Photosynthesis	Photosystem I	psaE	Photosystem I subunit IV, cp precursor	Bathy03g01910	contig03050	228	231	98.7%	contig03266	230	231	99.6%
Photosynthesis	Photosystem I	psaF	Photosystem I subunit III, cp precursor	Bathy06g03860	contig16018	643	663	97.0%	contig12124	655	663	98.8%
Photosynthesis	Photosystem I	psaG	Photosystem I subunit V	Bathy03g00890					contig02658	380	384	99.0%
Photosynthesis	Photosystem I	psaH	Photosystem I subunit VI, cp precursor	Bathy02g02090	contig05999	142	142	100.0%	contig24146	401	408	98.3%

Table S5

Category	Pathway	Gene	Gene Product	Gene in bathycoccus	T-142_contig	T-142_bases	T-142_bases similar	T-142_identity	T-149_contig	T-149_bases	T-149_bases similar	T-149_identity
Photosynthesis	Photosystem I	psaJ	Photosystem I subunit IX	BathyCg00125								
Photosynthesis	Photosystem I	psaK	Photosystem I subunit X, cp precursor	Bathy06g01380	contig01024	300	304	98.7%	contig00177	299	304	98.4%
Photosynthesis	Photosystem I	psaL	Photosystem I subunit XI, cp precursor	Bathy14g02590								
Photosynthesis	Photosystem I	psaN	Photosystem I subunit N, cp precursor	Bathy05g00070	contig01662	302	303	99.7%				
Photosynthesis	Photosystem I	ycf4	Photosystem I assembly protein ycf4	Bathy05g03790					contig02960	707	725	97.5%
Photosynthesis	Photosystem II	psbA	Photosystem II D1 protein	BathyCg00220					contig19783	133	133	100.0%
Photosynthesis	Photosystem II	ALB3.1	ALBINO3-like protein, cp precursor	Bathy09g02270	contig02316	1518	1533	99.0%	contig02971	1515	1533	98.8%
Photosynthesis	Photosystem II	ALB3.2	ALBINO3-like protein, cp precursor	Bathy03g01160					contig00947	755	762	99.1%
Photosynthesis	Photosystem II	ALB3.2	ALBINO3-like protein, cp precursor	Bathy03g01160					contig00946	501	513	97.7%
Photosynthesis	Photosystem II	PSBM	Photosystem II PSBM protein	Bathy16g00520					contig04736	366	373	98.1%
Photosynthesis	Photosystem II	PSBO	Photosystem II manganese-stabilizing polypeptide	Bathy04g04810	contig00901	723	728	99.3%	contig01614	548	550	99.6%
Photosynthesis	Photosystem II	PSBP	Photosystem II oxygen-evolving complex 23 kDa protein	Bathy12g01870	contig01643	597	601	99.3%	contig06285	167	172	97.1%
Photosynthesis	Photosystem II	PSBP	Photosystem II oxygen-evolving complex 23 kDa protein	Bathy12g01870					contig00378	598	601	99.5%
Photosynthesis	Photosystem II	PSBQ	Oxygen-evolving enhancer protein 3 (OEE3), cp precurso	Bathy16g01170	contig05212	174	174	100.0%	contig06199	374	377	99.2%
Photosynthesis	Photosystem II	PSBR	Photosystem II PSBR protein, cp precursor	Bathy03g05330	contig02413	277	279	99.3%	contig01190	277	279	99.3%
Photosynthesis	Photosystem II	PSBS	Photosystem II PSBS protein	Bathy04g01070	contig00330	778	795	97.9%	contig01199	537	538	99.8%
Photosynthesis	Photosystem II	PSBS	Photosystem II PSBS protein	Bathy04g01071	contig01861	206	229	90.0%	contig14940	241	260	92.7%
Photosynthesis	Photosystem II	PSBS	Photosystem II PSBS protein	Bathy04g01071					contig25805	229	261	87.7%
Photosynthesis	Photosystem II	PSBY	Photosystem II PSBY protein	Bathy07g04130					contig07165	311	329	94.5%
Photosynthesis	Soluble electron carriers and puta	FDX4	Chlamydomonas FDX4-like	Bathy05g03450	contig02583	416	428	97.2%	contig26239	327	328	99.7%
Photosynthesis	Soluble electron carriers and puta	FDX6	Chlamydomonas FDX6-like	Bathy05g01380	contig07715	430	434	99.1%	contig00492	279	281	99.3%
Photosynthesis	Soluble electron carriers and puta	FDX6	Chlamydomonas FDX6-like	Bathy05g01380					contig00495	369	370	99.7%
Photosynthesis	Soluble electron carriers and puta	PETF	Ferredoxin, chloroplast precursor	Bathy06g01620					contig06481	165	166	99.4%
Photosynthesis	Soluble electron carriers and puta	PETF	Ferredoxin, chloroplast precursor	Bathy06g01620					contig06482	134	134	100.0%
Photosynthesis	Soluble electron carriers and puta	PETF	Ferredoxin, chloroplast precursor	Bathy05g01380	contig07715	430	434	99.1%	contig00495	369	370	99.7%
Photosynthesis	Soluble electron carriers and puta	PETF	Ferredoxin, chloroplast precursor	Bathy05g01380					contig00492	279	281	99.3%
Photosynthesis	Soluble electron carriers and puta	PETF	Ferredoxin, chloroplast precursor	Bathy09g01240					contig05169	505	508	99.4%
Photosynthesis	Soluble electron carriers and puta	PETH	Ferredoxin-NADP oxidoreductase	Bathy09g00710	contig05806	480	480	100.0%	contig04474	1070	1073	99.7%
Photosynthesis	Soluble electron carriers and puta	PETJ	Cytochrome c553, chloroplast precursor	Bathy06g00630	contig01743	290	299	97.0%	contig00454	663	685	96.8%
Photosynthesis	Soluble electron carriers and puta	PETJ	Cytochrome c553, chloroplast precursor	Bathy06g00631	contig01738	233	241	96.7%				
**Photosynthesis**								**98.6%**				**98.6%**
Pigment synthesis	Carotenoid pathway	DXR	1-deoxy- D-xylulose-5-phosphate reductoisomerase	Bathy08g02640	contig04583	303	305	99.3%	contig02271	1259	1267	99.4%
Pigment synthesis	Carotenoid pathway	DXS	1-deoxy-D-xylulose-5-phosphate synthase	Bathy14g03060	contig15979	734	737	99.6%				
Pigment synthesis	Carotenoid pathway	HDS	1-hydroxy-2-methyl-2-(E)-butenyl-4-diphosphate synthas	Bathy10g03500	contig14405	239	248	96.4%	contig23724	1355	1370	98.9%
Pigment synthesis	Carotenoid pathway	HDS	1-hydroxy-2-methyl-2-(E)-butenyl-4-diphosphate synthas	Bathy10g03501	contig11070	457	465	98.3%	contig04616	861	867	99.3%
Pigment synthesis	Carotenoid pathway	HDS	1-hydroxy-2-methyl-2-(E)-butenyl-4-diphosphate synthas	Bathy10g03502	contig11160	314	315	99.7%				
Pigment synthesis	Carotenoid pathway	MCS	2-C-methyl-D-erythritol 2,4-cyclodiphosphate synthase	Bathy18g01440	contig08002	548	553	99.1%	contig08538	549	553	99.3%
Pigment synthesis	Carotenoid pathway	CMK	4-diphosphocytidyl-2-C-methyl-D-erythritol kinase	Bathy16g01460					contig00528	937	945	99.2%
Pigment synthesis	Carotenoid pathway	CMS	4-diphosphocytidyl-2-C-methyl-D-erythritol synthase	Bathy04g04020					contig11171	426	479	88.9%
Pigment synthesis	Carotenoid pathway	CMS	4-diphosphocytidyl-2-C-methyl-D-erythritol synthase	Bathy04g04020					contig02476	166	166	100.0%
Pigment synthesis	Carotenoid pathway	CYP97A	Carotene ß-hydrolase (cytochrome P450)	Bathy04g02170					contig02198	1827	1851	98.7%
Pigment synthesis	Carotenoid pathway	CRTH	Carotenoid isomerase	Bathy06g01280	contig02365	1730	1767	97.9%	contig04499	1255	1274	98.5%
Pigment synthesis	Carotenoid pathway	CRTH	Carotenoid isomerase	Bathy06g01281					contig04494	476	495	96.2%
Pigment synthesis	Carotenoid pathway	CRTH	Carotenoid isomerase	Bathy06g01282					contig04493	455	477	95.4%
Pigment synthesis	Carotenoid pathway	GGPS	Geranylgeranyl pyrophosphate synthase	Bathy07g03500	contig05810	774	774	100.0%	contig02244	1115	1131	98.6%
Pigment synthesis	Carotenoid pathway	GGR	Geranylgeranyl reductase	Bathy14g02760					contig16045	449	449	100.0%
Pigment synthesis	Carotenoid pathway	IDI	Isopentenyl diphosphate isomerase	Bathy08g04700					contig03814	817	819	99.8%
Pigment synthesis	Carotenoid pathway	PDS	Phytoene desaturase / phytoene dehydrogenase	Bathy11g01800	contig02545	123	149	82.6%	contig23449	1650	1685	97.9%
Pigment synthesis	Carotenoid pathway	PDS	Phytoene desaturase / phytoene dehydrogenase	Bathy11g01801	contig01243	1507	1550	97.2%	contig01748	144	177	81.4%
Pigment synthesis	Carotenoid pathway	PDS	Phytoene desaturase / phytoene dehydrogenase	Bathy11g01802	contig01270	127	136	93.4%				
Pigment synthesis	Carotenoid pathway	PSY1	Phytoene synthase / geranylgeranyldiphosphate geranylg	Bathy03g04230	contig02921	1228	1233	99.6%	contig04295	1225	1233	99.4%
Pigment synthesis	Carotenoid pathway	VDE	Violaxanthin deepoxidase	Bathy07g04400	contig03648	379	382	99.2%	contig24210	1435	1464	98.0%
Pigment synthesis	Carotenoid pathway	VDE	Violaxanthin deepoxidase	Bathy02g04870	contig08215	337	344	98.0%	contig00998	1407	1441	97.6%
Pigment synthesis	Carotenoid pathway	ZEP1	Zeaxanthin epoxidase	Bathy07g04390	contig01746	644	646	99.7%	contig02577	1565	1566	99.9%
Pigment synthesis	Carotenoid pathway	ZEP1	Zeaxanthin epoxidase	Bathy07g04390	contig10193	421	422	99.8%				
Pigment synthesis	Chlorophyll pathway	DVR	3,8-divinyl protochlorophyllide a 8-vinyl reductase	Bathy02g01050	contig00322	1162	1192	97.5%	contig03043	953	967	98.6%
Pigment synthesis	Chlorophyll pathway	CAO	Chlorophyll a oxygenase (chlorophyll b synthase)	Bathy07g03400	contig17322	280	287	97.6%	contig03458	217	238	91.2%

Table S5

Category	Pathway	Gene	Gene Product	Gene in bathycoccus	T-142_contig	T-142_bases	T-142_bases similar	T-142_identity	T-149_contig	T-149_bases	T-149_bases similar	T-149_identity
Pigment synthesis	Chlorophyll pathway	CAO	Chlorophyll a oxygenase (chlorophyll b synthase)	Bathy07g03400	contig17543	626	629	99.5%	contig02264	593	601	98.7%
Pigment synthesis	Chlorophyll pathway	CAO	Chlorophyll a oxygenase (chlorophyll b synthase)	Bathy07g03400	contig17419	331	339	97.6%	contig02242	580	605	95.9%
Pigment synthesis	Chlorophyll pathway	CAO	Chlorophyll a oxygenase (chlorophyll b synthase)	Bathy07g03400	contig02046	272	289	94.1%				
Pigment synthesis	Chlorophyll pathway	CHLG	Chlorophyll synthetase	Bathy04g01050	contig00330	1243	1281	97.0%	contig01197	217	238	91.2%
Pigment synthesis	Chlorophyll pathway	CHLG	Chlorophyll synthetase	Bathy04g01050					contig01195	578	583	99.1%
Pigment synthesis	Chlorophyll pathway	CHLG	Chlorophyll synthetase	Bathy04g01050					contig26428	449	454	98.9%
Pigment synthesis	Chlorophyll pathway	CHLG	Chlorophyll synthetase	Bathy04g01050					contig25148	448	453	98.9%
Pigment synthesis	Chlorophyll pathway	HEMF1	Coproporphyrinogen III oxidase	Bathy09g03310	contig16840	733	736	99.6%	contig02104	969	972	99.7%
Pigment synthesis	Chlorophyll pathway	HEMF1	Coproporphyrinogen III oxidase	Bathy09g03310	contig00513	376	397	94.7%	contig02105	186	192	96.9%
Pigment synthesis	Chlorophyll pathway	HEMF1	Coproporphyrinogen III oxidase	Bathy01g01060	contig03052	709	716	99.0%	contig26779	720	726	99.2%
Pigment synthesis	Chlorophyll pathway	HEMF1	Coproporphyrinogen III oxidase	Bathy01g01060	contig03051	563	572	98.4%	contig02978	416	419	99.3%
Pigment synthesis	Chlorophyll pathway	HEMF1	Coproporphyrinogen III oxidase	Bathy01g01060					contig02979	415	439	94.5%
Pigment synthesis	Chlorophyll pathway	HEMF1	Coproporphyrinogen III oxidase	Bathy01g01060					contig27761	297	298	99.7%
Pigment synthesis	Chlorophyll pathway	HEMF1	Coproporphyrinogen III oxidase	Bathy01g01060					contig27762	293	296	99.0%
Pigment synthesis	Chlorophyll pathway	HEMF1	Coproporphyrinogen III oxidase	Bathy01g01060					contig26780	308	342	90.1%
Pigment synthesis	Chlorophyll pathway	HEMF1	Coproporphyrinogen III oxidase	Bathy09g00170					contig09476	1115	1139	97.9%
Pigment synthesis	Chlorophyll pathway	HEMF1	Coproporphyrinogen III oxidase	Bathy09g00170					contig02538	605	614	98.5%
Pigment synthesis	Chlorophyll pathway	HEMB	Delta-aminolevulinic acid dehydratase	Bathy01g02350					contig02313	930	943	98.6%
Pigment synthesis	Chlorophyll pathway	HEMH	Ferrochelatase II	Bathy13g01310	contig03288	1187	1200	98.9%	contig23742	1191	1202	99.1%
Pigment synthesis	Chlorophyll pathway	HEML	Glutamate-1-semialdehyde 2,1-aminomutase	Bathy15g00080					contig07366	191	193	99.0%
Pigment synthesis	Chlorophyll pathway	GLURS	Glutamyl-tRNA synthetase	Bathy01g03590	contig00181	2215	2265	97.8%	contig00739	2214	2265	97.7%
Pigment synthesis	Chlorophyll pathway	HEMA	Glutamyl-tRNA reductase	Bathy13g02850	contig11203	154	157	98.1%	contig05030	503	507	99.2%
Pigment synthesis	Chlorophyll pathway	HEMA	Glutamyl-tRNA reductase	Bathy13g02850					contig05031	369	372	99.2%
Pigment synthesis	Chlorophyll pathway	HEMA	Glutamyl-tRNA reductase	Bathy13g02850					contig05028	319	322	99.1%
Pigment synthesis	Chlorophyll pathway	HEMA	Glutamyl-tRNA reductase	Bathy13g02850					contig05029	131	131	100.0%
Pigment synthesis	Chlorophyll pathway	HY1	Heme oxygenase	Bathy09g00140					contig04684	920	931	98.8%
Pigment synthesis	Chlorophyll pathway	POR1	Light-dependent protochlorophyllide oxidoreductase, cp p	Bathy02g03930	contig03860	734	739	99.3%	contig04432	1210	1218	99.3%
Pigment synthesis	Chlorophyll pathway	POR1	Light-dependent protochlorophyllide oxidoreductase, cp p	Bathy02g03930	contig03861	147	150	98.0%				
Pigment synthesis	Chlorophyll pathway	CHLM	Magnesium-protoporphyrin IX methyltransferase	Bathy02g03070	contig01572	959	966	99.3%	contig04027	951	984	96.6%
Pigment synthesis	Chlorophyll pathway	HEMC	Porphobilinogen deaminase	Bathy14g01900	contig15016	296	299	99.0%	contig10936	1007	1009	99.8%
Pigment synthesis	Chlorophyll pathway	HEMC	Porphobilinogen deaminase	Bathy14g01900	contig10679	252	253	99.6%	contig16459	117	119	98.3%
Pigment synthesis	Chlorophyll pathway	CHLD	Protoporphyrin IX Mg-chelatase subunit D	Bathy03g05660					contig18297	607	615	98.7%
Pigment synthesis	Chlorophyll pathway	CHLD	Protoporphyrin IX Mg-chelatase subunit D	Bathy03g05660					contig10554	868	878	98.9%
Pigment synthesis	Chlorophyll pathway	CHLD	Protoporphyrin IX Mg-chelatase subunit D	Bathy03g05660					contig11715	544	547	99.5%
Pigment synthesis	Chlorophyll pathway	CHLH	Protoporphyrin IX Mg-chelatase subunit H	Bathy06g04280	contig04924	1556	1567	99.3%	contig10023	526	533	98.7%
Pigment synthesis	Chlorophyll pathway	CHLH	Protoporphyrin IX Mg-chelatase subunit H	Bathy06g04280					contig12234	544	547	99.5%
Pigment synthesis	Chlorophyll pathway	CHLI	Protoporphyrin IX Mg-chelatase subunit I	Bathy15g01790	contig00650	1393	1413	98.6%	contig00886	1271	1286	98.8%
Pigment synthesis	Chlorophyll pathway	HEMG	Protoporphyrinogen IX oxidase	Bathy05g03760					contig07153	1251	1263	99.0%
Pigment synthesis	Chlorophyll pathway	HEMD	Urophorphyrin III synthase	Bathy07g03780	contig00603	1010	1056	95.6%	contig09968	701	729	96.2%
Pigment synthesis	Chlorophyll pathway	HEME1	Uroporphyrinogen III decarboxylase 1	Bathy07g02300					contig12172	914	920	99.3%
Pigment synthesis	Chlorophyll pathway	HEME2	Uroporphyrinogen III decarboxylase 2	Bathy06g01930					contig25416	1238	1248	99.2%
Pigment synthesis	Chlorophyll pathway	HEME3	Uroporphyrinogen III decarboxylase 3	Bathy01g04530	contig08079	1142	1151	99.2%	contig04564	1210	1231	98.3%
Pigment synthesis	Chlorophyll pathway	HEME4	Uroporphyrinogen III decarboxylase 4	Bathy03g01110	contig06488	775	786	98.6%	contig24442	692	705	98.2%
**Pigment synthesis**								**97.8%**				**97.8%**
Vitamin	Biotin or vitamin B7	bioB	Biotin synthase	Bathy11g00270	contig00531	1310	1314	99.7%	contig09240	1279	1283	99.7%
Vitamin	Biotin or vitamin B7	bioF	KAPA synthase	Bathy11g00060	contig11155	896	914	98.0%	contig02567	603	613	98.4%
Vitamin	Folate Biosynthesis	alpL	Alkaline phosphatase	Bathy11g02150	contig00465	1563	1618	96.6%	contig02965	1564	1618	96.7%
Vitamin	Folate Biosynthesis	dhfR	Dihydrofolate reductase	Bathy03g03500	contig06781	526	530	99.2%	contig26939	1534	1539	99.7%
Vitamin	Folate Biosynthesis	dhfS	Dihydrofolate synthase	Bathy10g02810					contig02060	1633	1668	97.9%
Vitamin	Folate Biosynthesis	dhpS	Dihydropteroate synthase	Bathy05g03140	contig08788	376	382	98.4%	contig05810	1827	1875	97.4%
Vitamin	Folate Biosynthesis	dhpS	Dihydropteroate synthase	Bathy05g03140	contig08792	230	232	99.1%				
Vitamin	Folate Biosynthesis	gch1	GTP cyclohydrolase I	Bathy03g00180	contig11317	403	410	98.3%	contig09955	2087	2121	98.4%
Vitamin	Folate Biosynthesis	gch1	GTP cyclohydrolase I	Bathy03g00180	contig13121	538	539	99.8%				
Vitamin	Folate Biosynthesis	gch1	GTP cyclohydrolase I	Bathy03g00180	contig13132	266	266	100.0%				
**Vitamin**								**98.5%**				**98.3%**
