# Supplementary material for: Metagenomes of the Picoalga Bathycoccus from the Chile Coastal Upwelling
Source: PLoS One. 2012 Jun 22;7(6):e39648. doi: 10.1371/journal.pone.0039648 (PMC3382182; doi:10.1371/journal.pone.0039648)
Supplement: Table S6 — Estimation of the number of major haplotypes and of their frequency for selected regions of genes with a high read coverage in both samples. (PDF) [file pone.0039648.s010.pdf]

Table S6

| Gene code | Gene name | T142 | T149 | T142 & T149 | T142 # reads | | | | | | T149 # reads | | | | | | T142 % of each genotype | | | | | | T149 % of each genotype | | | | | |
|---|---|---|---|---|---|---|---|---|---|---|---|---|---|---|---|---|---|---|---|---|---|---|---|---|---|---|---|---|
| | | # of major genotype | | # of genotype in common | geno 1 | geno 2 | geno 3 | geno 4 | geno 5 | other | geno 1 | geno 2 | geno 3 | geno 4 | geno 5 | other | geno 1 | geno 2 | geno 3 | geno 4 | geno 5 | other | geno 1 | geno 2 | geno 3 | geno 4 | geno 5 | other |
| Bathy01g02150 | Cleavage factor | 2 | 3 | 2 | 17 | 3 | | | | | 30 | 15 | 9 | | | | 85% | 15% | | | | | 56% | 28% | 17% | | | |
| Bathy02g01050 | dvr1 (Pigment synthesis) | 2 | 2 | 2 | 22 | 4 | | | | | 48 | 34 | | | | | 85% | 15% | | | | | 59% | 41% | | | | |
| Bathy02g03140 | cdc23 | 2 | 3 | 2 | 110 | 23 | | | | | 14 | 3 | 4 | | | | 83% | 17% | | | | | 67% | 14% | 19% | | | |
| Bathy02g01210 | ABC transporter | 2 | 3 | 0 | 379 | 6 | | | | | | | 21 | 11 | 6 | 2 | 98% | 2% | | | | | | | 53% | 28% | 15% | 5% |
| Bathy02g01270 | Multicopper oxydase type 2 | 1 | 2 | 1 | 860 | | | | | | 13 | 17 | | | | 1 | 100% | | | | | | 42% | 55% | | | | 3% |
| Bathy02g05100 | Hypothetical | 3 | 3 | 3 | 13 | 6 | 5 | | | | 60 | 1 | 1 | | | 1 | 54% | 25% | 21% | | | | 95% | 2% | 2% | | | 2% |
| Bathy02g05160 | Hypothetical | 3 | 1 | 0 | 15 | 11 | 3 | | | | | | | 134 | 9 | 7 | 52% | 38% | 10% | | | | | | | 89% | 6% | 5% |
| Bathy03g03310 | Hypothetical | 2 | 3 | 1 | 12 | 17 | | | | | 24 | | 14 | 10 | | | 41% | 59% | | | | | 50% | | 29% | 21% | | |
| Bathy03g02910 | Hypothetical | 3 | 2 | 1 | 20 | 17 | 2 | | | | | | 16 | 2 | | 2 | 51% | 44% | 5% | | | | | | 80% | 10% | | 10% |
| Bathy04g01080 | Hypothetical | 1 | 2 | 0 | 24 | | | | | 1 | | 14 | 19 | | | | 96% | | | | | 4% | | 42% | 58% | | | |
| Bathy04g01140 | Hypothetical | 3 | 2 | 2 | 5 | 7 | 2 | | | | 13 | 17 | | | | | 36% | 50% | 14% | | | | 43% | 57% | | | | |
