# Supplementary material for: Metagenomes of the Picoalga Bathycoccus from the Chile Coastal Upwelling
Source: PLoS One. 2012 Jun 22;7(6):e39648. doi: 10.1371/journal.pone.0039648 (PMC3382182; doi:10.1371/journal.pone.0039648)
Supplement: Table S7 — Genotype variability within the 5,646 bp rRNA operon. Positions are given along chromosome 11. Localisation of helix 3 of ITS2 follows Marin and Melkonian [39]. (PDF) [file pone.0039648.s011.pdf]

Table S7

Localisatio	Gene	RCC1105	T142				T149			
chromo_11			Genotype 1		Genotype 2		Genotype 1		Genotype 2	
		Base	Base	# of reads	Base	# of reads	Base	# of reads	Base	# of reads
123 885	18S rRNA	**T**	**T**	3	C	12				
122 368	ITS2 rRNA	**T**	**T**	2	A	11	**T**	3	A	2
122 507	ITS2 rRNA	**T**					**T**	2	C	3
123 039	ITS2 rRNA	**A**	**A**	14	G	3				
Note : Helix 3 is located between position 122 384 and 122 486										
