# Supplementary material for: Metagenomes of the Picoalga Bathycoccus from the Chile Coastal Upwelling
Source: PLoS One. 2012 Jun 22;7(6):e39648. doi: 10.1371/journal.pone.0039648 (PMC3382182; doi:10.1371/journal.pone.0039648)
Supplement: Table S8 — Assignment of reads for Pacific picoeukaryote samples T142 to individual chromosomes of O.’ lucimarinus’ using Geneious Assembler (see Materials and Methods for details). (PDF) [file pone.0039648.s012.pdf]

Table S8

		T142	T142	T142	T142	T142
	Length	Reads	Coverage	Coverage	Coverage Depth	Identical sites
**Chromosome**	**bp**	**#**	**bp**	**%**	**x**	**%**
Ostre_luci_chrom01	1 152 508	108	13 495	1.10%	0.01	99.90%
Ostre_luci_chrom02	895 087	119	5 560	0.50%	0.01	99.80%
Ostre_luci_chrom03	982 987	175	10 579	1.00%	0.04	99.80%
Ostre_luci_chrom04	930 724	93	18 883	2.00%	0.02	99.90%
Ostre_luci_chrom05	847 696	55	8 174	0.90%	0.01	99.90%
Ostre_luci_chrom06	818 664	50	5 954	0.60%	0.01	99.90%
Ostre_luci_chrom07	783 246	120	22 947	2.90%	0.04	99.90%
Ostre_luci_chrom08	701 771	260	31 421	4.40%	0.12	99.80%
Ostre_luci_chrom09	670 853	61	12 726	1.80%	0.02	99.80%
Ostre_luci_chrom10	613 585	43	7 367	1.10%	0.01	99.90%
Ostre_luci_chrom11	593 542	33	3 438	0.50%	0.01	99.90%
Ostre_luci_chrom12	538 963	189	9 675	1.60%	0.10	99.70%
Ostre_luci_chrom13	528 469	30	4 232	0.70%	0.01	99.90%
Ostre_luci_chrom14	708 927	50	4 275	0.50%	0.01	99.90%
Ostre_luci_chrom15	468 366	48	4 672	0.90%	0.01	99.80%
Ostre_luci_chrom16	428 333	72	16 029	3.70%	0.04	99.90%
Ostre_luci_chrom17	366 173	47	5 447	1.30%	0.02	99.80%
Ostre_luci_chrom18	149 386	6	357	0.10%	0.00	99.90%
Ostre_luci_chrom19	154 676	13	676	0.20%	0.00	99.70%
Ostre_luci_chrom20	549 133	53	7 835	1.30%	0.02	99.90%
Ostre_luci_chrom21	321 799	15	2 184	0.60%	0.01	99.90%
**Total or mean**	13 204 888	1 640	195 926	1.48%		
