# Supplementary material for: Metagenomes of the Picoalga Bathycoccus from the Chile Coastal Upwelling
Source: PLoS One. 2012 Jun 22;7(6):e39648. doi: 10.1371/journal.pone.0039648 (PMC3382182; doi:10.1371/journal.pone.0039648)
Supplement: Table S9 — List of reference genomes used for BLASTX analysis of contigs. (PDF) [file pone.0039648.s013.pdf]

Table S9

**Taxonmic group**	**Genome type**	**Species and genome version**	**Strain**	**Number of proteins**	**GenBank / Link**
Mamiellophyceae	Nuclear	*Ostreococcus* RCC809 2.0	RCC809	7 492	http://genome.jgi-psf.org/OstRCC8
Mamiellophyceae	Nuclear	*Ostreococcus lucimarinus* v2.0	CCE9901	7 651	NC_009355-75
Mamiellophyceae	Nuclear	*Bathycoccus prasinos*	RCC1105	8 747	http://bioinformatics.psb.ugent.be/
Mamiellophyceae	Nuclear	*Micromonas pusilla* CCMP1545 v2.0	CCMP1545	10 547	ACCP00000000
Mamiellophyceae	Nuclear	*Micromonas* RCC299 v3.0	RCC299	10 109	NC_013038-54
Mamiellophyceae	Nuclear	*Ostreococcus tauri*	OTTH0595	7 890	CR954201-20
Haptophyta	Nuclear	*Emiliania huxleyi* v1.0	CCMP1516	39 125	http://genome.jgi-psf.org/Emihu1/E
Stramenopiles	Nuclear	Fragilariopsis cylindrus model 1		27 137	http://genome.jgi-psf.org/Fracy1/Fr
Stramenopiles	Nuclear	*Phaeodactylum tricornutum* v2.0	CCAP 1055/1	10 025	NC_011669-701
Stramenopiles	Nuclear	*Thalassiosira pseudonana* v3.0	CCMP1335	11 390	NC_012064-87
Stramenopiles	Nuclear	*Aureococcus anophagefferens*	CCMP1984	11 501	http://genome.jgi-psf.org/Auran1/A
Fungi	Nuclear	*Lachancea (Saccharomyces) kluyveri*	CBS3082	5 328	CM000687-94
Fungi	Nuclear	*Candida glabrata*	CBS138	5 202	NC_005967-36
Mamiellophyceae	Mitochondrion	*Micromonas* RCC299 v3.0	RCC299	39	NC_012643
Mamiellophyceae	Mitochondrion	*Ostreococcus tauri*	OTTTH0595	43	CR954200
Rhodophyta	Mitochondrion	*Cyanidioschyzon merolae*	10D	34	NC_000887
Mamiellophyceae	Chloroplast	*Micromonas* RCC299 v3.0	RCC299	57	NC_012575
Mamiellophyceae	Chloroplast	*Ostreococcus tauri*	OTTTH0595	61	CR954199
Rhodophyta	Chloroplast	*Cyanidioschyzon merolae*	10D	207	NC_004799
Bacteria		*Roseobacter denitrificans*	OCh 114	3 946	NC_008209
Bacteria		*Candidatus* Pelagibacter ubique	HTCC1062	1 354	NC_007205
Bacteria		*Synechococcus* sp.	CC9902	2 206	NC_007513
Bacteria		*Prochlorococcus marinus*	MIT 9313	2 269	NC_005071
Bacteria		*Bacillus* sp.	B14905	4 624	NZ_AAXV00000000
Bacteria		*Marinobacter aquaeolei*	VT8	3 858	NC_008740
Bacteria		*Planctomyces maris*	DSM 8797	6 480	NZ_ABCE00000000
Archaea		*Thermococcus onnurineus*	NA1	1 975	NC_011529
Archaea		*Halomicrobium mukohataei*	DSM 12286	3 173	NC_013202
Virus (prasinovirus)		*Ostreococcus tauri* virus OtV5		243	NC_010191
Virus (prasinovirus)		*Ostreococcus tauri* virus OtV1		240	NC_013288
Virus (prasinovirus)		*Bathycoccus prasinos* virus BpV161		218	NC_014765
Virus (prasinovirus)		*Bathycoccus prasinos* virus BpV115		211	HM004430
Virus (prasinovirus)		*Ostreococcus lucimarinus* virus OlV1		251	NC_014766
Virus (prasinovirus)		*Micromonas pusilla* virus MpV1		244	NC_014767
Virus		*Acanthamoeba polyphaga* mimivirus		911	NC_04649
Virus		*Emiliania huxleyi* virus 86		472	NC_007346
